# Dynamic allostery in the peptide/MHC complex enables TCR neoantigen selectivity

**DOI:** 10.21203/rs.3.rs-4457195/v1

**Published:** 2024-05-29

**Authors:** Jiaqi Ma, Cory M. Ayres, Chad A. Brambley, Smita S. Chandran, Tatiana J. Rosales, Steven A. Corcelli, Evgenii L. Kovrigin, Christopher A. Klebanoff, Brian M. Baker

**Affiliations:** 1. Department of Chemistry and Biochemistry, University of Notre Dame, Notre Dame, IN, USA; 2. Harper Cancer Research Institute, University of Notre Dame, Notre Dame, IN, USA.; 3. Human Oncology and Pathogenesis Program, Memorial Sloan Kettering Cancer Center (MSKCC), New York, NY, USA.; 4. Center for Cell Engineering, MSKCC, New York, NY, USA.; 5. Weill Cornell Medical College, New York, NY, USA.; 6. Parker Institute for Cancer Immunotherapy, New York, NY, USA.

## Abstract

The inherent cross-reactivity of the T cell receptor (TCR) is balanced by high specificity, which often manifests in confounding ways not easily interpretable from static structures. We show here that TCR discrimination between an HLA-A*03:01 (HLA-A3)-restricted public neoantigen derived from mutant *PIK3CA* and its wild-type (WT) counterpart emerges from motions within the HLA binding groove that vary with the identity of the peptide’s first primary anchor. The motions form a dynamic gate that in the complex with the WT peptide impedes a large conformational change required for TCR binding. The more rigid neoantigen is insusceptible to this limiting dynamic, and with the gate open, is able to transit its central tryptophan residue underneath the peptide backbone to the contralateral side of the HLA-A3 peptide binding groove, facilitating TCR binding. Our findings reveal a novel mechanism driving TCR specificity for a cancer neoantigen that is rooted in the dynamic and allosteric nature of peptide/MHC-I complexes, with implications for resolving long-standing and often confounding questions about the determinants of T cell specificity.

## Introduction

Using their T cell receptor (TCR), T cells orchestrate cellular immunity by recognizing antigenic peptides bound and presented by major histocompatibility complex (MHC) proteins. Cross-reactivity is a hallmark of TCRs, ensuring a TCR repertoire limited to millions of clonotypes can accommodate a vastly larger array of potential ligands (1, 2). Paradoxically however, TCRs are highly specific, and can show surprising sensitivity to subtle modifications to antigenic peptides. Although specificity can often be interpreted in the context of static structural features, such as amino acid substitutions that alter hot spots in the TCR-peptide/MHC interface or alter the conformation of the peptide in the MHC binding groove (3), in many cases, TCR sensitivity to peptide modifications cannot be readily interpretable from structural information alone. This has particularly been the case with peptides presented by class I MHC proteins, with examples of “structurally silent” TCR specificity dating back to the earliest days of structural immunology (4).

More recently, we described another striking example of structurally silent TCR specificity that elevated its implications. Studying the gp100_209_ shared tumor antigen presented by HLA-A*02:01 (HLA-A2), we found that different TCRs distinguished between the identity of the side chain of the position 2 primary anchor residue: replacing the sub-optimal threonine at position 2 of the peptide with methionine weakened the binding of the gp100_209_/HLA-A2-specific TCR SILv44, but enhanced the binding of the gp100_209_/HLA-A2-specific TCR T4H2, despite no perceivable differences in the static crystallographic structures of the peptide/MHC complexes (5). These results were all the more striking as the side chains of position 2 residues in class I MHC-presented peptides are accommodated in the deep “B” pocket of the binding groove, inaccessible to direct contact by TCRs (6). Remarkably, the SILv44 and T4H2 TCRs were able to distinguish between methionine and norleucine at position 2 of gp100_209_, which differ only by the presence of a sulfur instead of a methylene carbon within the side chain. Notably, position 2-modified variants of the gp100209 peptide have been widely studied as potential heteroclitic cancer vaccines but have failed to achieve significant clinical benefit (7), in part because the anchor modifications render the WT and modified peptides antigenically distinct in this structurally silent fashion (8). Observations similar to those with the gp100_209_ tumor antigen have been made with anchor-modified variants of other tumor antigens (5, 9–13).

In addition to heteroclitic immunogens, structurally silent TCR specificity has implications for the design of personalized neoantigen vaccines using *in silico* prediction tools. Prediction efforts for such neoantigens typically compare the properties of the mutant epitope to its wild-type (WT) counterpart, and in many cases emphasize mutations at primary anchor positions. Notably, vaccine predictions using current algorithms are routinely characterized by high rates of both false positives and false negatives (14).

We recently described a panel of TCRs that recognize a public neoantigen resulting from a hotspot mutation in the phosphoinositide 3-kinase p110α catalytic subunit (PI3Kα, encoded by *PIK3CA*) that is presented by HLA-A*03:01 (HLA-A3) (15). The neoantigen results from substitution of a leucine for histidine at the first primary anchor (position 2; sequence A**H**HGGWTTK → A**L**HGGWTTK). The mutation enhances peptide binding to HLA-A3 and the neoantigen drives T cell destruction of *PIK3CA* mutant tumors while leaving tumor cells expressing wild-type *PIK3CA* unharmed. Although the crystallographic structures of the neoantigen and WT peptide/HLA-A3 complexes were nearly indistinguishable, we were unable to detect functional recognition of the WT peptide/HLA-A3 complex, despite the WT peptide binding HLA-A3 strongly enough that, based on comparative data from other systems (16–18), responses should have been measurable in the presence of typical TCR cross-reactivity. We concluded this was likely another example of structurally silent TCR specificity.

Our work with the *PIK3CA* neoantigen provides an opportunity to study structurally silent TCR specificity in the context of a shared neoantigen with immediate clinical relevance. Using multiple orthogonal techniques, including X-ray crystallography, molecular dynamics simulations, and nuclear magnetic resonance (NMR) spectroscopy, we show here that TCR recognition of the neoantigen/HLA-A3 complex is critically dependent on a large conformational change in the center of the peptide, in which the tryptophan at position 6 (pTrp6) flips from aligning against the HLA-A3 α1 helix to aligning against the α2 helix. The conformational change, among the largest documented for TCR binding, is promoted by higher frequency intrinsic dynamics in the peptide and proceeds through a surprising motional pathway which sees the Trp6 side chain rotate underneath and around the axis of the peptide backbone. Compared to the neoantigen/HLA-A3 complex, the complex with the WT peptide samples a different and broader conformational ensemble. Via a gating mechanism that involves both the peptide and the HLA-A3 protein, the motional landscape in the WT complex hinders the conformational change required for TCR binding. These results directly illustrate the presence of dynamic allostery, in which protein motions propagating from changes at one site influence functionally important motions at a distant site (19, 20). In this case, dynamic allostery in the peptide/HLA-A3 complex explains structurally silent TCR specificity and enables selectivity for a clinically relevant cancer neoantigen.

Beyond the implications for *PIK3CA* neoantigen based immunotherapy, our results help rationalize how TCRs can be inherently cross-reactive and simultaneously highly specific. They highlight the dynamic and allosteric nature of peptide/MHC complexes and emphasize how the properties of the peptide/HLA ligand are enmeshed with the properties of the receptor in determining T cell specificity. Such dynamic properties are not always apparent from static structures but are likely a key feature of cellular immunity, with significant implications for T cell specificity and the biological principles and therapeutic design efforts that depend on it, including the identification of immunogenic cancer neoantigens (21, 22).

## Results

### TCRs distinguish between the *PIK3CA* neoantigen and WT peptide despite nearly identical static structures

Our previous work indicated that, although T cell responses could clearly be observed against the *PIK3CA* neoantigen, multiple neoantigen-specific TCRs were unable to recognize the WT peptide/HLA-A3 complex (15). Even though less potency is expected from the WT peptide given its weaker binding to HLA-A3, in other instances where peptides modified at the first primary anchor have been examined, weaker binding peptides have still elicited quantifiable functional responses (16, 23, 24). This is exemplified by studies with the MART-1 shared tumor antigen, where MHC binding affinities for the native nonamer (AAGIGILTV) and anchor-modified decamer (ELAGIGILTV) are nearly the same as the *PIK3CA* WT peptide and neoantigen (18). Yet despite its weaker binding, measurable responses to the native MART-1 nonamer have been described for numerous T cell clones (16, 17).

**Figure 1.**
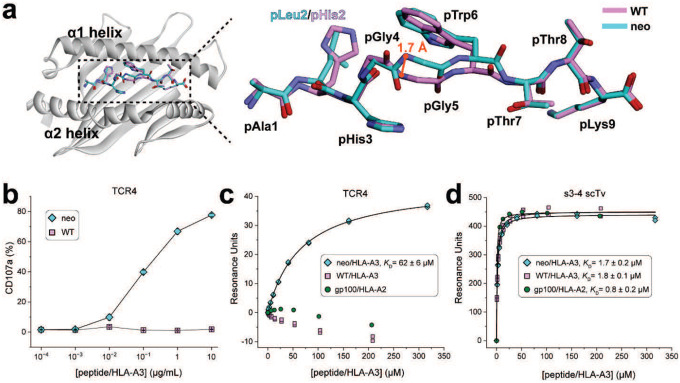
TCRs distinguish between the *PIK3CA* neoantigen and WT peptide presented by HLA-A3 despite overlapping structures. **A)** Comparison of the crystallographic structures of the neoantigen and WT peptide in the HLA-A3 binding groove, demonstrating the overall structural similarities. A small divergence is seen in the backbone regions between pGly4 and pTrp6, likely reflecting intrinsic peptide dynamics. The neoantigen is in cyan, the WT peptide in pink; this color scheme is maintained throughout all figures. Superimposition is by all common peptide atoms, with an RMSD of 0.7 Å. **B)** Measurement of T cell function via the degranulation marker CD107a. T cells expressing the neoantigen-specific receptor TCR4 were co-cultured with HLA-A3^+^ antigen presenting cells in the presence of increasing amounts of neoantigen or WT peptide. Although neoantigen recognition is clear, there is no recognition of the WT peptide. Data shown are absolute frequencies derived from the averages of two independent experiments with three biological replicates per experiment. Points are means and error bars are standard deviations. **C)** SPR experiments of TCR4 with the neoantigen or WT peptide/HLA-A3 complexes. Although neoantigen recognition was quantifiable, no binding was detected with the WT peptide. gp100/HLA-A2 is an irrelevant negative control complex of the peptide IMDQVPFSV presented by HLA-A2, for which no binding was also detected. The *K*_D_ and error for neoantigen binding are the average and standard deviation of six replicates. **D)** The neoantigen and WT peptide/HLA-A3 complexes bind the s3–4 scTv with identical affinities, indicating the WT sample is stable over the course of an SPR experiment, indicating our inability to detect TCR binding to the WT peptide/HLA-A3 is due to TCR discrimination. The gp100/HLA-A2 control was also recognized well. *K*_D_ and error values are the average and standard deviation of three replicates. Titrations in panels B-D were performed at 4 °C for increased peptide/MHC stability.

The inability to detect T cell responses against the WT peptide was curious given our structural work with the neoantigen and WT peptide/HLA-A3 complexes that showed nearly identical conformations for the peptides in the binding groove (15). Although we observed a slight 1–2 Å variance in the central backbone resulting from φ/ψ bond differences in the glycines at positions 4 and 5 and hypothesized this could underlie specificity, the differences in the conformations of the peptides in these static structures are within the error limits recently established by analyses of the structures of replicate class I peptide/MHC complexes (all atom common root mean square deviation [RMSD] of 0.7 Å, compared to 0.7 ± 0.4 Å for the analysis of 30 replicate nonameric class I peptide/MHC structures) (25) (Fig. 1A). Moreover, as discussed below, in structures with two, unrelated neoantigen/HLA-A3-specific TCRs (TCR3 and TCR4), the central region of the peptide undergoes a large conformational change upon TCR binding. Altogether, this led us to hypothesize that TCR specificity for the *PIK3CA* neoantigen emerges from intrinsic peptide dynamics within the binding groove.

We thus sought to resolve the mechanistic basis for how *PIK3CA* neoantigen-specific TCRs can achieve selectivity for the mutant over the WT peptide. To confirm the inability of T cells to recognize the WT peptide, we first examined stimulation of T cells transduced with TCR4, among the most sensitive of our previously described TCRs. Co-culture experiments with HLA-A3^+^ antigen presenting cells measuring the degranulation marker CD107a showed a clear dose response curve for the neoantigen, with an EC_50_ value near 100 nM (Fig. 1B). However, no stimulation was evident with the WT peptide, even at peptide concentrations as high as 10 μM.

To verify the functional experiments biochemically, we generated recombinant TCR4 and assessed its binding to both the neoantigen and WT peptide/HLA-A3 complexes in a direct binding experiment that allowed us to control for the weaker binding of the WT peptide. We coupled TCR4 to one flowcell of a surface plasmon resonance (SPR) sensor surface. In an adjacent flowcell, we coupled the recently described single chain TCR variant (scTv) s3–4. As s3–4 binds class I MHC proteins away from the peptide binding groove with an affinity independent of the bound peptide (26), this reagent allowed us to verify the integrity of the WT peptide/HLA-A3 complex during the experiment. To help ensure the stability of the samples, we also performed the experiments at the reduced temperature of 4 °C.

As before, binding to the neoantigen complex was readily detected, with the experiments yielding a *K*_D_ of 62 ± 6 μM for TCR4 binding the neoantigen/HLA-A3 complex (Fig. 1C). No binding was detectable with the WT complex. However, strong binding to the s3–4 scTv positive control was apparent with both peptide/HLA-A3 samples, with the experiments yielding a *K*_D_ of 1.7 ± 0.2 μM for the neoantigen complex and an identical *K*_D_ of 1.8 ± 0.1 μM for the WT complex (Fig. 1D). Thus, our inability to detect TCR binding to the WT peptide/HLA-A3 complex is attributable to the ability of the TCR to distinguish between the neoantigen and the WT peptide.

### TCR recognition of the neoantigen is critically dependent on the rotation of the central tryptophan residue

**Figure 2.**
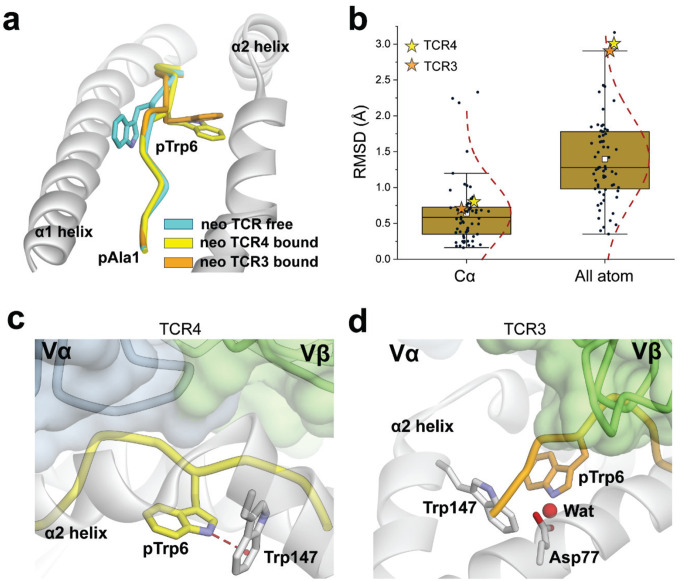
TCR binding to the *PIK3CA* neoantigen induces a large conformational change in the peptide that leads to new peptide-HLA interactions. **A)** Illustration of the peptide conformational change that occurs upon the binding of TCR3 and TCR4 to the neoantigen/HLA-A3 complex. The tryptophan at position 6 has flipped from aligning against the α1 helix to nestling between the peptide backbone and the α2 helix. **B)** Distribution of conformational changes that occur in nonameric class I MHC complexes upon TCR binding as measured by bound/free RMSDs for Cα atoms and all peptide atoms. The values for the *PIK3CA* neoantigen upon recognition by TCR3 or TCR4 are shown as orange and yellow stars, respectively. The white square in the box plot gives the average, the gold box the interquartile range (IQR), and the whiskers 1.5×IQR. Although the changes for the backbone are only slightly above the mean, when considering all peptide atoms, the conformational change in the neoantigen is among the largest seen upon TCR recognition of nonamers. **C)** In the complex with TCR4, the pTrp6 side chain is not contacted by the TCR. The indole nitrogen of the flipped conformation of pTrp6 forms a hydrogen bond with Trp147 of the HLA-A3 α2 helix. **D)** In the complex with TCR3, the pTrp6 side chain is also not contacted by the TCR. The slight repositioning of the pTrp6 side chain in the complex with TCR3 distorts the interaction of pTrp6 with Trp147, although a structural water in close proximity bridges pTrp6 to Asp77 of the α1 helix.

The *PIK3CA* neoantigen is notable in that, upon recognition by multiple TCRs, the peptide undergoes a large conformational change. In the free (i.e., not TCR-bound) peptide/HLA-A3 complex, the side chain of the tryptophan at position 6 (pTrp6) packs against the HLA-A3 α1 helix, whereas in the structures with two unique neoantigen-specific TCRs (TCR3 and TCR4), the side chain and the neighboring backbone units have flipped around the axis of the peptide backbone, with the pTrp6 side chain buried within the groove and packed against the α2 helix (Fig. 2A). When all atoms of the peptide are considered, the conformational change (referred to as a ‘flip’ for pTrp6) is among the largest yet observed for TCRs binding nonameric peptides presented by class I MHC proteins (Fig. 2B).

While the flip of pTrp6 is a defining structural feature of *PIK3CA* neoantigen recognition by multiple TCRs, its role in TCR recognition is not clear, as the flipped side chain is not contacted in the complexes with either TCR3 or TCR4. In the structure with TCR4, the indole nitrogen of the side chain forms a hydrogen bond with the aromatic ring of tryptophan 147 of HLA-A3 (Fig. 2C). The slight change in the position of the side chain in the complex with TCR3 distorts the geometry of this interaction, although the side chain is still embedded in a complex electrostatic environment with structural water linking the indole nitrogen to the HLA-A3 α1 helix (Fig. 2D). Substitution of pTrp6 in the neoantigen with alanine or glycine eliminates functional recognition with both TCRs (15); however, neither of these substitutions probes the peptide conformational change.

**Figure 3.**
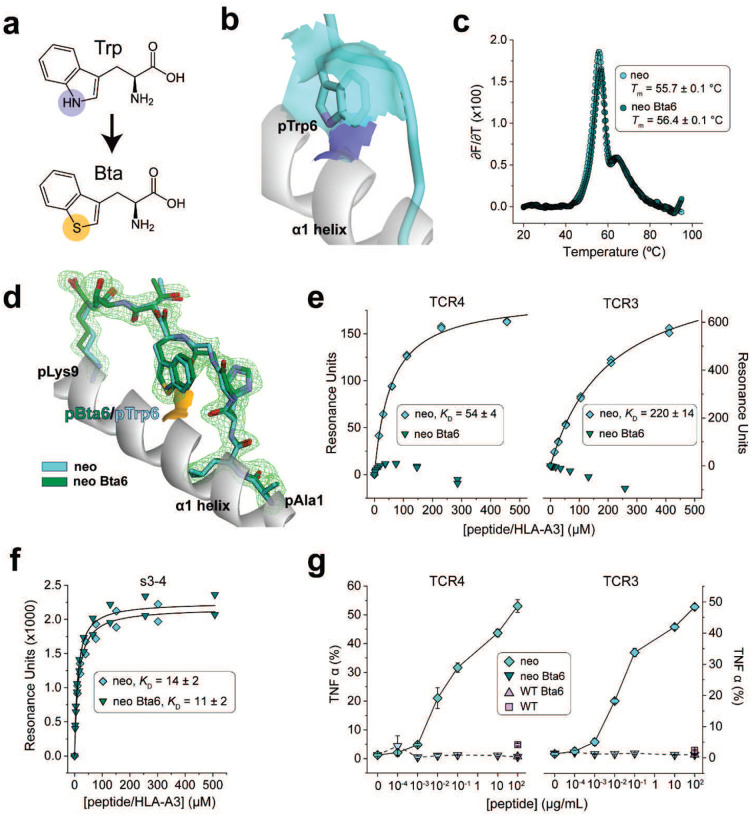
TCR recognition of the *PIK3CA* neoantigen presented by HLA-A3 is critically dependent on the flip of the tryptophan at position 6. **A)** The tryptophan analog Bta replaces the indole NH with a sulfur atom, removing the capacity of the tryptophan side chain to serve as a hydrogen bond donor. **B)** In the structure of the free neoantigen/HLA-A3 complex, the pTrp6 side chain remains accessible to solvent. The accessible surface of the indole nitrogen is blue; the surface of the carbon atoms is cyan. **C)** Substitution of pTrp6 with Bta does not alter peptide binding to HLA-A3 as indicated by differential scanning fluorimetry. Datapoints indicate the temperature derivative of the fluorescence ratio; only every 5^th^ datapoint is shown for clarity. *T*_m_ and error values are the average and standard deviation of four replicates. **D)** Substitution of pTrp6 with Bta does not alter the structural properties of the neoantigen in the HLA-A3 binding groove as shown by the crystallographic structure of the Bta6-neoantigen/HLA-A3 complex. The Bta-substituted peptide is superimposed on the unsubstituted neoantigen, with an all-common atom RMSD of 0.7 Å. The orange surface shows the solvent accessibility of the Bta sulfur atom to compare with that of the indole nitrogen in panel B. Electron density of the Bta-substituted neoantigen is from a 2F_o_-F_c_ composite OMIT map calculated with simulated annealing, contoured at 1σ. **E)** SPR experiments show no detectable binding of TCR4 or TCR3 to the Bta-substituted neoantigen/HLA-A3 complex, although binding to the non-substituted neoantigen complex was quantifiable. Experiments were performed at 25 °C; *K*_D_ and error values are the average and standard deviation of three replicates. **F)** SPR experiments confirming binding of the Bta-substiuted and non-substituted neoantigen/HLA-A3 complexes to the peptide-independent s3–4 scTv. Experiments were performed at 25 °C; *K*_D_ and error values are the average and standard deviation of four replicates. **G)** Measurement of T cell function via production of the cytokine TNF-α. T cells expressing either TCR4 or TCR3 were co-cultured with HLA-A3^+^ antigen presenting cells in the presence of increasing amounts of Bta6-modified or unmodified neoantigen. Although unmodified neoantigen recognition is clear, there is no recognition of the Bta6-modified version. The unmodified and Bta6-modified WT peptides at the highest concentration were included as controls. Data shown are absolute frequencies derived from the averages of two independent experiments with three biological replicates per experiment. Points are means and error bars are standard deviations.

We thus designed an experiment to directly probe the importance of the neoantigen conformational change. The tryptophan analog 3-benzothienyl-L-alanine (Bta) is isomorphic with tryptophan but replaces the indole nitrogen with a sulfur atom incapable of serving as a hydrogen bond donor (Fig. 3A). We reasoned that removing the hydrogen bond donor would destabilize the TCR-bound flipped peptide conformation, with a destabilized conformation (or, equivalently, higher energy state) leading to weaker TCR binding. At the same time, removing the hydrogen bond donor by substituting pTrp6 with Bta should have little to no impact on the TCR-free conformation, as in the neoantigen/HLA-A3 structure, the side chain at position 6 remains accessible to solvent (Fig. 3B). We verified that substitution of pTrp6 with Bta did not impact the binding of the peptide to HLA-A3 using differential scanning fluorimetry: as expected, there was no impact on the stability of the peptide/HLA-A3 complex (Fig. 3C). We next determined the crystallographic structure of HLA-A3 presenting the Bta-substituted peptide (Supplemental Table 1) The structure showed there were no significant changes in peptide conformation, with the Bta6 side chain aligned against the HLA-A3 α1 helix and the sulfur atom solvent exposed, mimicking the conformation of the neoantigen (Fig. 3D). All common atoms of the neoantigen and Bta-substituted variant superimposed with an RMSD of 0.6 Å.

We assessed the recognition of the Bta-substituted neoantigen biochemically and functionally. Using SPR we could not detect binding of TCR4 or TCR3 to the complex of HLA-A3 with the Bta-substituted neoantigen (Fig. 3E), although this complex was readily recognized by the s3–4 scTv positive control (Fig. 3F). Functionally, the Bta-substituted neoantigen also failed to drive cytokine secretion by T cells transduced with TCR3 or TCR4 when co-cultured with HLA-A3^+^ antigen presenting cells, although the un-modified neoantigen control was well-recognized (Fig. 3G). TCR recognition of the *PIK3CA* neoantigen is thus critically dependent on the flip of pTrp6.

### Conformational sampling of pTrp6 differs between the neoantigen and WT peptide

**Figure 4.**
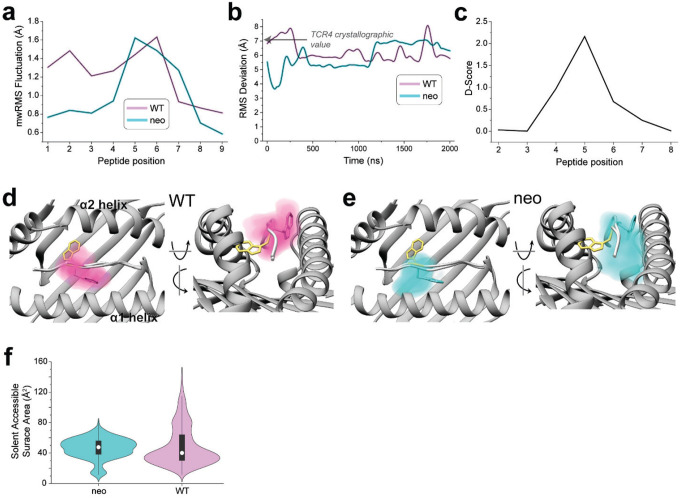
Conformational sampling differs in the *PIK3CA* neoantigen and WT peptide/HLA-A3 complexes. **A)** Mass-weighted RMS fluctuations for each amino acid of the neoantigen and WT peptide in the HLA-A3 binding groove from 2 μs of unrestrained, fully atomistic molecular dynamics simulations. The central regions of both peptides are mobile, as is the N-terminal half of the WT peptide but not the neoantigen, consistent with the neoantigen’s more optimal position 2 anchor. **B)** Despite high mobility, neither peptide samples the TCR-bound conformation, as shown by the RMSD of the pTrp6 amino acid relative to its position in the ternary complex with TCR4 during the simulations (data were smoothed using LOWESS; see Extended Data Fig. 7A for unsmoothed data). The value of 7.1 Å from superimposition of the TCR4-bound and free structures is indicated by the arrow. **C)** Conformational sampling differs across the centers of the peptide backbones of the neoantigen and WT peptide as indicated by a D-score analysis comparing average φ/ψ bond angles during the two simulations. **D)** Conformational space occupied by the pTrp6 side chain during the simulation with the WT peptide. Color density reflects degree of sampling (voxels sampled <10% of the time excluded). Note the tendency for the pTrp6 side chain to move above the peptide backbone; although it reaches over, it does not flip to the TCR-bound state as shown in panel B. **E)** As in panel D, except volume occupied by pTrp6 during the simulation with the neoantigen. Note the tendency for the pTrp6 side chain to move under the peptide backbone. **F)** Solvent accessible surface areas of the pTrp6 side chain during the neoantigen and WT simulations. Although the average values are similar as indicated by the central white circles of the violin plot, the WT peptide samples a much wider range of values, reflecting the volume analyses in panels D and E.

Conformational changes that occur upon binding are often reflected in the motions of the unbound protein (27). To investigate these, as well as assess any differences in the dynamics of the *PIK3CA* neoantigen and WT peptide bound to HLA-A3, we studied the motions of the peptides with a series of computational simulations. We first performed fully atomistic, unrestrained molecular dynamics (MD) on the peptide/HLA-A3 complexes, simulating each complex for a lengthy 2 μs in explicit solvent. We examined peptide motion by computing full atom, mass-weighted root mean square fluctuations (mwRMSFs) for each amino acid of the two peptides. The neoantigen showed high fluctuations in its central bulge (positions 4 to 7) but was more rigid at both termini (Fig. 4A). The WT peptide showed similar high fluctuations in the center, but also had high fluctuations in its N-terminal region, consistent with its sub-optimal position 2 anchor of histidine (as opposed to leucine in the neoantigen).

As high fluctuations were seen in the centers of both peptides, we next asked if either the neoantigen or WT peptide sampled the flipped conformation. For both trajectories, we computed the RMSD of pTrp6 from its conformation in the TCR4 ternary complex, selecting this metric due to TCR4’s higher affinity and potency compared to TCR3. The RMSD analysis indicated that, despite the high fluctuations, neither peptide sampled the TCR-bound state during the simulations (Fig. 4B). Interestingly though, this analysis also suggested that conformational sampling differed between the two peptides. To further investigate, we analyzed the peptides during the two trajectories using a metric referred to as the D-score, which was developed to evaluate conformational properties of antibody loops and more recently used to describe the conformations of peptides in class I MHC binding grooves (28, 29). The D-score provides a convenient way to compare differences in backbone torsion angles, and ranges from 0 when two amino acids have identical φ/ψ bond angles to 8 when both angles differ by 180°. When the neoantigen and WT peptide were compared this way, the average D-score was low at the termini, but peaked in regions from positions 4 to 7 (Fig. 4C). Thus, although the neoantigen and WT peptide show similarly high fluctuations across their centers, their conformational ensembles differ.

To visualize the space sampled by the centers of the neoantigen and WT peptides, we defined 3D grids centered around pTrp6 of the neoantigen and WT peptide, with a spacing of 0.1 Å. We then tabulated the fractional occupancy of each resulting voxel by the atoms of pTrp6. As expected from the fluctuation data, we observed disperse sampling for both the neoantigen and WT peptides (Figs. 4D,E). Consistent with the D-score analysis though, sampling was different between the two peptides. The tryptophan side chain in the WT peptide remained close to its crystallographic conformation but also sampled states above the binding groove that left the side chain largely solvent exposed (Fig. 4D). In contrast, the side chain in the neoantigen sampled states more recessed in the binding groove, including states which placed the side chain underneath the peptide bulge (Fig. 4E).

**Extended Data Figure 1.**
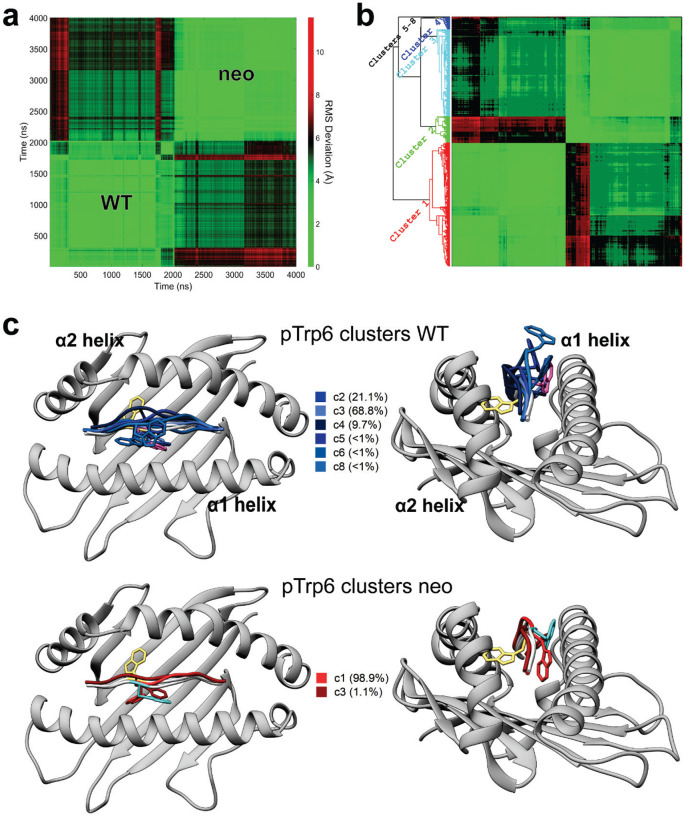
Conformational clustering reveals different conformational states sampled by pTrp6 in the *PIK3CA* neoantigen and WT peptide/HLA-A3 complexes. **A)** 2D RMSD analysis of the pTrp6 amino acid over the course of the neoantigen and WT peptide/HLA-A3 simulations after superimposition of the HLA-A3 binding groove. Quadrants for the WT and neoantigen simulations are indicated; the much higher values in the cross-simulation quadrants illustrate the different conformational sampling. **B)** Cluster analysis of the 2D RMSD data from panel A (supplemented with TCR-free and TCR4-bound coordinates as indicated in the Methods). pTrp6 clustered into 8 major conformations as indicated. Cluster 2 reflected the TCR-free and Cluster 7 (not sampled during the simulations) reflected the TCR4-bound conformations. **C)** Visualization of the pTrp6 conformational clusters for the WT peptide (top) and neoantigen (bottom), showing the tendency for pTrp6 to move above the backbone in the WT simulation, but below the backbone in the neoantigen simulation. The crystallographic coordinates of the WT, TCR-free neoantigen, and TCR4-bound neoantigen are colored magenta, cyan, and yellow.

To examine individual conformations adopted, we calculated pairwise (or two-dimensional) RMSDs of pTrp6 in the two simulations, as well as the initial free and TCR4-bound crystallographic coordinates, following superimposition of the peptide binding grooves (Extended Data Fig. 1A). The results clustered into eight distinct conformations (Extended Data Figs. 1B,C), with the WT peptide sampling six of those conformations and the neoantigen sampling two. Only one conformation (cluster 3) was sampled by both peptides, and the coordinates of the TCR4-bound neoantigen (cluster 7) were not sampled at all during the simulations. In agreement with the conformational clustering analysis, the greater conformational variability for the pTrp6 side chain in the WT peptide is further reflected in an analysis of the side chain’s solvent accessible surface area during the two simulations, which shows similar average levels of accessibility, but a much wider range for the WT peptide vs the neoantigen (Fig. 4F).

### Different conformational sampling of pTrp6 emerges more extensive position 2 motions in the WT complex

**Extended Data Figure 2.**
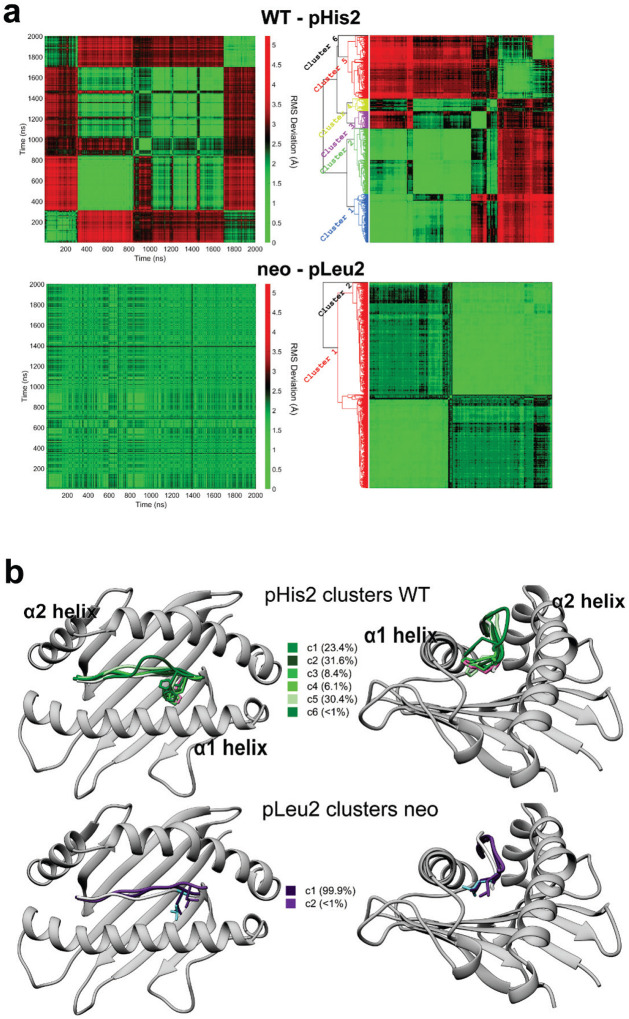
Conformational clustering illustrates the significant dynamic differences between the position 2 amino acid in the neoantigen and WT peptide. **A)** 2D RMSD analysis and conformational clustering for pHis2 in the WT simulation (top) and pLeu2 in the neoantigen simulation (bottom), illustrating the substantially greater conformational diversity for the position 2 anchor in the WT vs. neoantigen. **B)** Visualization of the position 2 conformational clusters for the WT peptide (top) and neoantigen (bottom), further illustrating the more dynamic behavior of position 2 in the WT peptide. The crystallographic coordinates of the WT and TCR-free neoantigen are colored magenta and cyan.

**Figure 5.**
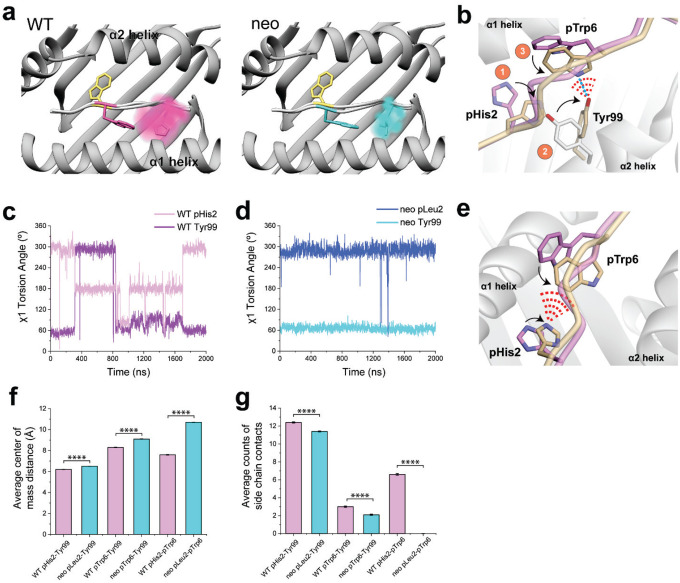
The extensive motions of p2His in the WT peptide/HLA-A3 complex lead to more direct and indirect interactions with p6Trp as it moves in the binding groove. **A)** Volume occupied by pHis2 during the simulations with the WT peptide (left) or pLeu2 during the simulations with the neoantigen (right). Color density reflects degree of sampling (voxels sampled <10% of the time excluded). Note the greater mobility of pHis2 in the WT peptide. The TCR4-bound conformation of pTrp6 is colored yellow. **B)** In the simulation with the WT peptide, the movement of pHis2 induces an alternate conformation for Tyr99 of HLA-A3 and subsequent formation of contacts (red dashes) and a hydrogen bond (blue line) between the Tyr99 altered conformer and pTrp6 in the WT peptide. Structural snapshot is representative of cluster 1 in Fig. S2B. **C)** The χ1 torsion angles of Tyr99 of HLA-A3 and pHis2 of the peptide during the WT peptide/HLA-A3 simulation. During the first half of the simulation, the rotation in pHis2 induces a rotation in Tyr99 (in the latter half of the simulation, the peptide N-terminus has become less recessed in the binding groove, decoupling pHis2/Tyr99 motion). **D)** The χ1 torsion angles of pLeu2 and Tyr99 remain fixed in the simulation with the neoantigen. **E)** Other conformations of pHis2 in the simulation with the WT peptide show contacts (red dashes) between the side chains of the histidine and pTrp6. Structural snapshot is representative of cluster 2 in Extended Data Fig. 2. **F)** Average centers of mass between the side chains of the position 2 amino acid, pTrp6, and Tyr99 during the simulations with the neoantigen and WT peptide. Average distances are all closer in the WT simulation. Error bars are SEM, calculated from the 2000 1 ns frames of the 2 μs simulations. **** = p<0.0001. **G)** Average counts of side chain contacts between the side chains of the position 2 amino acid, pTrp6, and Tyr99 during the simulations with the neoantigen and WT peptide. Contacts are all higher in the WT simulation. Error bars are SEM, calculated from the 2000 1ns frames of the 2 μs simulations. **** = p<0.0001.

The differential dynamics in the N-terminal halves of the neoantigen and WT peptide revealed by the RMSF data (Fig. 4A) prompted us to examine conformations adopted by peptide position 2. We thus performed the same visualization analysis for position 2 that we performed for pTrp6. Consistent with the RMSF data, the suboptimal position 2 histidine of the WT peptide (p2His) was highly mobile, sampling a large volume within the HLA-A3 B pocket, whereas the position 2 leucine of the neoantigen (p2Leu) was tightly constrained (Fig. 5A). To examine the conformations sampled by these amino acids, we performed pairwise RMSD and clustering analyses as on pTrp6, but now on position 2. The more dynamic pHis2 of the WT peptide clustered into six conformations, compared to just two for the rigid pLeu2 of the neoantigen (Extended Data Figs. 2A,B). The predominant cluster of pLeu2 of the neoantigen was essentially identical to that of the crystallographically observed state. In contrast, the clusters for the more mobile pHis2 of the WT peptide had various alterations of the side chain’s χ1 and χ2 angles.

**Extended Data Figure 3.**
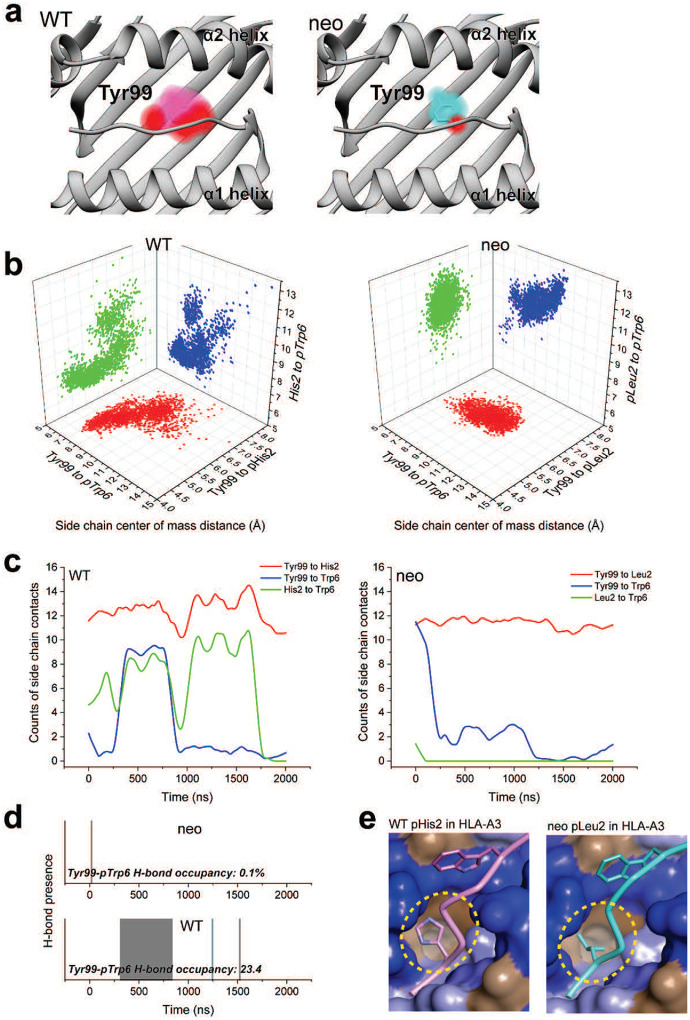
Side chain chains move closer and form more extensive interactions with pTrp6 in the WT compared to neoantigen simulations. **A)** Conformational space occupied by the HLA-A3 Tyr99 side chain during the simulations with the WT peptide (left) and neoantigen (right). Color density reflects degree of sampling (voxels sampled <10% of the time excluded). The red space is attributable to the tyrosine hydroxyl. Substantially greater space is sampled in the WT compared to the neoantigen simulation. **B)** Inter-side chain distances from the peptide/HLA-A3 simulations for pHis2/pLeu2, pTrp6, and Tyr99 of HLA-A3, measured by distances between side chain centers of mass. Points are for each ns of the 2 μs simulations. Data for the WT peptide are on the left; data for the neoantigen are on the right. The three side chains are more dynamic and move closer to each other in the WT compared to the neoantigen simulation. **C)** Counts of side chain-side chain contacts for pHis2/pLeu2, pTrp6, and Tyr99 of HLA-A3 as a function of time during the two simulations. More contacts are made in the WT simulation (left), particularly between peptide position 2 and pTrp6 and Tyr99 and pTrp6 (data were smoothed using LOWESS; see Extended Data Fig. 7B for unsmoothed data). **D)** Formation and persistence of the pTrp6-Tyr99 hydrogen bond in the neoantigen (top) and WT (bottom) simulations. The percentage of time the hydrogen bond exists is indicated; in the neoantigen simulation (top) it is essentially non-existent at 0.1%; whereas in the WT simulation (bottom) the hydrogen bond is present for 23% of the time. **E)** Visualization of how pLeu2 in the WT peptide and pHis2 in the neoantigen fit into the HLA-A3 B pocket in the respective crystallographic structures. pLeu2 of the neoantigen reaches further into pocket, whereas pHis2 lies in the depression. Surface is colored according to amino acid hydrophobicity, from blue (less hydrophobic) to brown (more hydrophobic).

Examining the p2His clusters of the WT peptide in more detail, we observed that one of the clusters with an alternate pHis2 position showed a large rotation in the χ1 torsion angle of the neighboring Tyr99 in the floor of the HLA-A3 binding groove, with the Tyr99 side chain rotating approximately 140° to avoid a steric clash with the histidine (cluster 1, with a population of 23%). This rotation placed Tyr99 directly under the bulge of the peptide backbone and adjacent to pTrp6, which had entered the base of the groove (Fig. 5B). Interrogating the WT simulation revealed a correlation between the rotation of χ1 of Tyr99 and pHis2 (Fig. 5C). Moreover, the rotation of Tyr99 was slightly (~1ns) preceded by rotation of pHis2, indicating a pathway of motion connecting pHis2 and Tyr99 to pTrp6. In contrast, in the simulation with the neoantigen, the χ1 torsion angles of pLeu2 and Tyr99 did not deviate from their initial positions (Fig. 5D). Visualization of the space sampled by Tyr99 as performed for pTrp6 and pHis2/pLeu2 confirmed it was highly mobile in the WT simulation, but rigid in the neoantigen simulation (Extended Data Fig. 3A).

We examined the other conformational clusters for p2His in the WT simulation and found that a majority (total population of simulation time of 63%) placed the histidine adjacent to pTrp6 as it entered the base of the groove (Fig. 5E). Together with the Tyr99 data, this prompted us to examine the distances between the position 2 side chain and the side chains of pTrp6, and Tyr99 in the two simulations. The comparison confirmed that not only were pHis2 and Tyr99 more dynamic in the WT simulation, but compared to the neoantigen simulation, they were on average closer in proximity to pTrp6 (Figs. 5F, Extended Data 3B). The closer proximity in the WT simulation led to the formation of substantial contacts between pHis2, pTrp6, and Tyr99 (Figs. 5G, Extended Data 3C), and even the formation of a pTrp6-Tyr99 hydrogen bond, which persisted for nearly a quarter of the simulation time (Figs. 5B, Extended Data 3D). However, such inter-residue interactions were reduced or, for the pLeu2-pTrp6 contacts or the Tyr99-pTrp6 hydrogen bond, essentially absent in the neoantigen simulation. The overall picture is that, for the WT peptide, the greater motion of the pHis2 side chain leads to numerous direct and indirect inter-residue interactions with pTrp6 as it enters the base of the binding groove. However, these interactions are far less significant for the neoantigen, permitting pTrp6 to reach further into base of the HLA-A3 peptide binding groove as shown in Fig. 4E.

We next asked why the pHis2 side chain in the wild-type peptide was so mobile in the HLA-A3 B pocket compared to the leucine of the neoantigen. HLA-A3 shows a strong preference for hydrophobic amino acids in its hydrophobic B pocket (30, 31). However, the deeper part of the pocket, into which pLeu2 of the neoantigen extends, is preceded by a hydrophobic depression. In the structure with the wild-type peptide, pHis2 lies in this depression (Extended Data Fig. 3E). From the structure of the wild-type peptide/HLA-A3 complex, we computed the *pK*_a_ of pHis2 of the wild-type peptide using continuum electrostatics (32). This resulted in an estimated *pK*_a_ of 4.9, which as expected would leave the histidine uncharged at physiologic pH values. However, the computed *pK*_a_ for pHis2 in the free peptide was 6.1, indicating an electrostatic destabilization of 1.6 kcal/mol. The resulting compression of the energy differences between conformational states translates into a flatter energy landscape and thus more rapid conformational interconversion, as has been seen in other cases when polar or charged amino acids are buried in hydrophobic environments (33).

### The flip in the neoantigen occurs with a high energy peptide-limbo mechanism

**Extended Data Figure 4.**
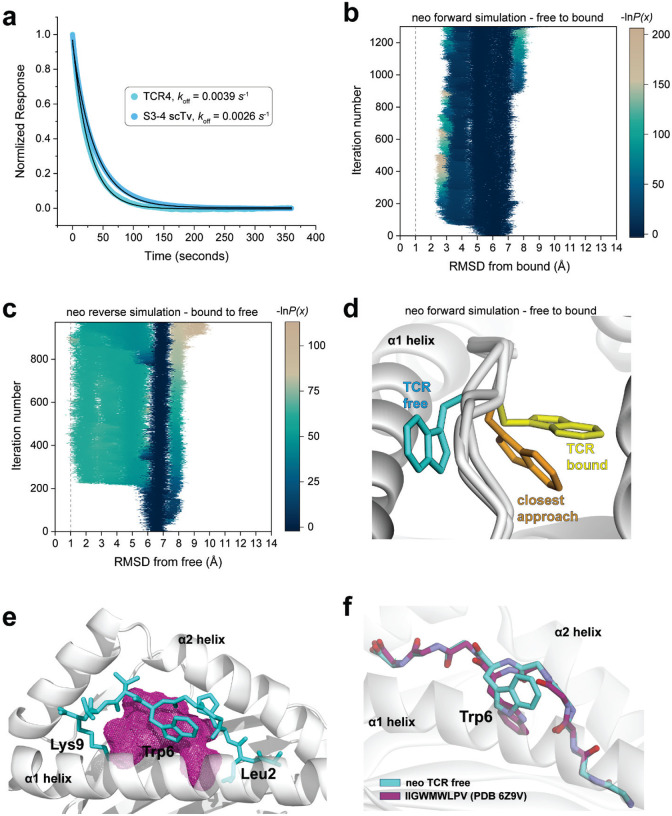
Correlates with the under-peptide flip mechanism in the *PIK3CA* neoantigen. **A)** Dissociation phases from SPR kinetic experiments, showing the dissociation rates for TCR4 and the s3–4 scTv from the neoantigen/HLA-A3 complex. Solid lines are fits to single exponential decay functions, with the *k*_off_ values indicated. Together with the *K*_D_ values in Fig. 1, the dissociation rates provide the association rates via *k*_on_ = *k*_off_/*K*_D_. **B)** Evolution of the neoantigen/HLA-A3 WEMD simulations in the forward direction, showing probability as a function of iteration number and RMSD from the target, in this case the conformation of pTrp6 in the ternary complex with TCR4 after starting from the TCR-free conformation. Over 1300 WEMD iterations, pTrp6 comes close to but does not reach the TCR-bound conformation. A successful transition was described as an RMSD < 1 Å, indicated by the dashed line. **C)** As in panel B, but for a reverse simulation, showing probability vs. iteration and the RMSD of pTrp6 from the TCR-free conformation after starting from the conformation in the TCR4 ternary complex. Over 970 WEMD iterations, a successful transition was observed 109 times. **D)** Closest approach of pTrp6 to its TCR4 bound conformation in the forward WEMD simulations, indicating that even though a successful transition was not observed, the side chain still traversed under the peptide backbone to the adjacent side of the binding groove. RMSD to bound is 2.4 Å. **E)** The large ~400 Å^3^ cavity under the peptide backbone in the neoantigen/HLA-A3 complex. **F)** Example of a peptide presented by HLA-A2 with a backbone conformation almost identical to that of the *PIK3CA* neoantigen bound to HLA-A3 and with pTrp6 pointing down into the base of the binding groove, resembling a snapshot midway through the neoantigen transition.

Our inability to observe the flip of the Trp6 side chain in the *PIK3CA* neoantigen during traditional MD simulations suggests a high energy barrier. To test this, we measured the association rate (*k*_on_) for binding of TCR4 to the neoantigen/HLA-A3 complex. We performed this by first measuring the dissociation (*k*_off_) rate for TCR4. Using the same SPR configuration we used to measure binding affinity in Fig. 1C, we determined a *k*_off_ of 0.039 ± 0.006 *s*^−1^ (Extended Data Fig. 4A). Using the relationship *k*_on_ = *k*_off_/*K*_D_, this yielded a *k*_on_ of 629 ± 14 M^−1^ *s*^−1^. Compared to other TCRs, this is an exceptionally slow association rate (34). For example, under the same conditions, the prototypical anti-viral TCR A6 binds the HTLV-1 Tax peptide presented by HLA-A2 approximately 20-fold faster, with a *k*_on_ of 1.1 × 10^4^ M^−1^ *s*^−1^ (35). Notably, the binding of A6 to Tax/HLA-A2 also occurs with a conformational change in the peptide backbone as well as conformational changes in both TCR CDR3 loops (36–38). Repeating the experiment but with the side-binding scTv s3–4, we measured a 25-fold faster *k*_on_ of 1.5 × 10^4^ M^−1^ *s*^−1^. Although multiple factors influence rates of protein association, the rates of conformational changes are a major contributor (39, 40). The very slow *k*_on_ for the binding of TCR4 is thus consistent with a high barrier for the flip of pTrp6 in the neoantigen.

To better study how the peptide crosses this barrier and moves to the TCR-bound state, we used weighted ensemble molecular dynamics simulations (WEMD) to identify potential motional pathways. Rather than spending simulation time sampling around stable states and waiting for low probability transitions, WEMD relies on multiple independent simulations run in parallel and tracks the progression of each simulation towards a defined target state. To overcome high barriers and access rare events, WEMD dynamically reweights trajectories based on their progress towards the target state, thereby sampling rare events without introducing biases such as steering forces (41, 42). We performed WEMD beginning with the neoantigen/HLA-A3 complex either in its TCR-free conformation (a forward simulation) or in its conformation in the ternary complex with TCR4 (a reverse simulation, performed with the TCR removed). Target states were defined as the crystallographic structures of the TCR4-neoantigen/HLA-A3 ternary complex (for the forward simulation) or the neoantigen/HLA-A3 complex (for the reverse simulation). The progress of the simulation was tracked by RMSD of pTrp6 between the trajectory and the target state. An RMSD < 1 Å was defined as a successful transition.

**Figure 6.**
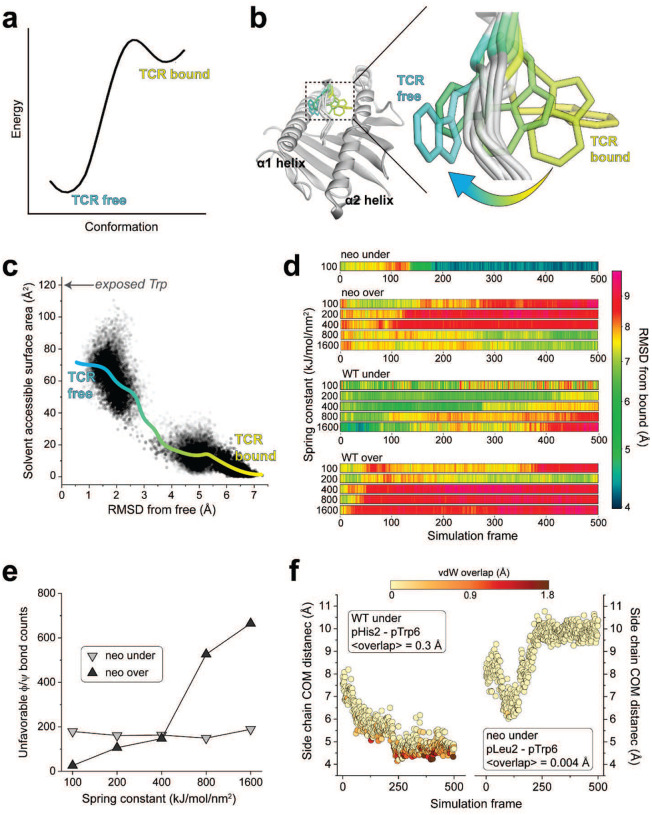
The flip in the *PIK3CA* neoantigen occurs via an under-peptide motional pathway. **A)** Energy diagram describing the peptide conformational change as a transition from a lower to higher energy flipped state, resulting in a high energy barrier in the forward direction and a low barrier in the reverse direction. **B)** Under-peptide motional pathway illuminated by the reverse WEMD simulations beginning with the conformation in the ternary complex with TCR4, showing pTrp6 of the neoantigen moving underneath the peptide backbone. The three under-peptide conformations are extracted from three roughly equally spaced timepoints of a single successful transition. **C)** Solvent accessible surface area of the pTrp6 side chain from each frame of the 109 successful reverse WEMD transitions. The solid cyan-to-yellow curve was generated from LOWESS smoothing of the data. The value expected from an exposed side chain if pTrp6 transitioned by moving over the top of the peptide (~120 Å^2^) is indicated by the arrow. **D)** RMSD of pTrp6 from the TCR4-bound conformation in SMD simulations of the peptide/HLA-A3 complexes forcing under- or over-peptide rotations. Data are shown for both the neoantigen and the WT peptide as a function of progressively larger spring constants. Only the neoantigen in an under-peptide rotation reaches the bound state, as indicated by the top row. **E)** Number of unfavored φ/ψ torsion angles for all non-terminal amino acids of the neoantigen in the under- or over-peptide SMD simulations, excluding glycines. Attempting to force an over-peptide rotation by increasing the spring constant results in greater torsional resistance. **F)** Distance between the center of mass (COM) of the pTrp6 side chain and the position 2 side chain in the WT peptide (left) or neoantigen (right) under-peptide SMD simulations, colored by degree of van der Waals (vdW) overlap between the side chains. In the neoantigen simulation, the side chains remain distant, with little to no atomic overlap. In the WT peptide simulation, the side chains come in close proximity, with substantial overlap as the simulation progresses. Data are from the simulations with the 100 kJ/mol/mm^2^ spring constant. Bracketed values give the average atomic overlap in Å.

While the forward simulation approached but did not fully flip even after 1300 iterations, we found 109 trajectories containing successful transitions over 970 iterations in the reverse simulation (Extended Data Figs. 4B,C). These results are consistent with a high energy barrier in the forward direction and a lower barrier in reverse (Fig. 6A). Remarkably, each of the successful trajectories from the reverse simulation showed the pTrp6 side chain moving underneath the peptide backbone, evoking a limbo dance (Fig. 6B; Supplemental Movie). This was evident not only from visual inspection, but also quantitatively from an analysis of the solvent accessible surface area of pTrp6 during the transitions, which increased as the peptide moved from its TCR-bound to TCR-free conformation but did not approach the value of an exposed amino acid tryptophan oriented above the peptide bulge (Fig. 6C). Additionally, even though we did not observe a complete transition in the forward simulations, the trajectory that most closely approached the target placed the pTrp6 sidechain underneath and on the other side of the peptide backbone near to its position in the TCR-bound state (Extended Data Fig. 4D). While a “peptide limbo” transition was initially surprising, the pathway revealed evokes the conformations seen in the traditional MD analysis of the neoantigen, which as noted above did not fully flip but did place the pTrp6 side chain further in the base of the groove (Fig. 4E).

The under-peptide transition suggests the presence of empty space between the peptide backbone and the floor of the HLA-A3 protein. To examine this, we computed open volumes (or cavities) in the neoantigen/HLA-A3 structure. This analysis revealed a large cavity with a volume of approximately 400 Å^3^ between the peptide and the HLA-A3 binding groove floor (Extended Data Fig. 4E). As the volume of tryptophan is approximately 160 Å^3^, the space needed to accommodate the motional pathway indicated by WEMD is clearly available. We examined other peptide/MHC complexes in the HLA-A3 superfamily whose structures were available and determined that cavities between the peptide backbone and floor of the groove are common, as the analysis of 36 different structures revealed an average cavity size of 178 Å^3^ with a large standard deviation of 113 Å^3^ (Supplemental Table 2). We also surveyed other structures of nonameric peptides bound to HLA-A proteins and found several examples of peptides with large position 6 side chains positioned under the peptide backbone. The structure of the peptide IIGWMWIPV bound to HLA-A2 is a notable example: the conformation of the peptide backbone is nearly identical to that of the neoantigen, yet the tryptophan at position 6 is oriented down towards the base of the groove (43), resembling an intermediate for the under-peptide transition of the neoantigen (Extended Data Fig. 4F). The architecture of the HLA-A3 peptide binding groove is thus clearly compatible with an under-peptide, limbo dance pathway.

**Extended Data Figure 5.**
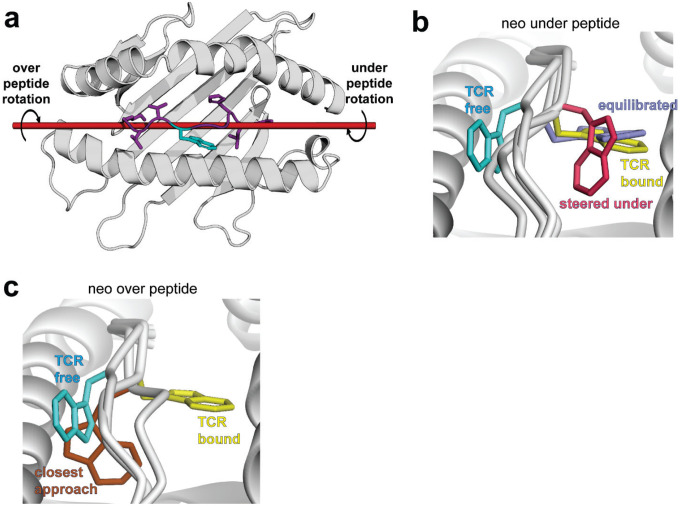
Steered molecular dynamics comparing the under-peptide with the over-peptide transition. **A)** Diagram showing the axis and directions for under- and. over-peptide enforced rotation performed by SMD. The pTrp6 amino acid was defined as the rotation group, with the axis of rotation set as a line through the peptide backbone as indicated. **B)** Closest approach of the pTrp6 side chain to the TCR4-bound state for the SMD under-peptide rotation (RMSD to bound is 3.7 Å, achieved with a spring constant of 100 kJ/mol/mm^2^). Unrestrained traditional molecular dynamics simulations on the neoantigen/HLA-A3 complex starting from the closest approach resulted in pTrp6 adopting the TCR4-bound state within 35 ns of simulation time (final RMSD to bound is 0.7 Å; indicated as equilibrated). **C)** Closest approach of the pTrp6 side chain to the TCR4-bound state for the SMD over-peptide rotation (RMSD to bound is 5.8 Å, achieved with a spring constant of 800 kJ/mol/mm^2^).

To check the validity of the under-peptide pathway, we performed steered molecular dynamics (SMD) to rotate the pTrp6 side chain under the backbone. SMD differs from WEMD in that, rather than searching for a probable pathway of motion, a pathway is stipulated through applied force. We used an approach termed enforced rotation, in which a set of atoms (defined as the rotation group) are pulled around a user-defined axis at a constant angular velocity via harmonic potentials, while still permitting motional relaxation and other movements throughout the protein and solvent as in a traditional simulation (44). Beginning with the structure of the neoantigen bound to HLA-A3, we assigned all pTrp6 atoms to the rotation group and defined a rotational axis as a vector through the peptide backbone (Extended Data Fig. 5A). With an initial spring constant of 100 kJ/mol/nm^2^, we first rotated pTrp6 in the neoantigen complex underneath the peptide backbone. We readily observed under-peptide rotation, with the pTrp6 side chain adopting a conformation near that seen in the structure with TCR4 within 500 ps of enforced rotation (Fig. 6D, **neo under**). Subsequent traditional MD initiated from this state resulted in the side chain equilibrating rapidly into a conformation nearly identical to that in the TCR4 ternary complex (Extended Data Fig. 5B).

As an alternative to rotating the pTrp6 side chain under the peptide backbone, we inverted the directionality of the rotational axis to rotate the side chain over the peptide backbone, attempting to force a trajectory which would leave the side chain solvent exposed along the pathway. Unlike the under-peptide case, in these simulations the peptide would not move into a TCR-bound state even with spring constants as high as 1600 kJ/mol/nm^2^ (Fig. 6D, **neo over**). The closest conformation reached still possessed an RMSD more than 6 Å from its conformation in the TCR4 ternary complex (Extended Data Fig. 5C). In addressing why there was no successful transition, we asked whether over-peptide rotation might be torsionally unfavored due to constraints on peptide φ/ψ bond angles. We computed the φ/ψ torsion angles along the peptide backbone for both over- and under-peptide rotations and for all spring constants simulated, noting any instance of an unfavored torsion (i.e., φ/ψ angles outside of the allowed and generously allowed regions of the Ramachandran plot for all non-terminal residues of the peptide, excluding glycine). For the over-peptide simulations, we found that torsional strain increased with increasing values of the spring constant: during the simulations, the number of unfavored φ/ψ torsions increased dramatically as the spring constant was raised from 100 to 1600 kJ/mol/nm^2^, even though no successful transition was observed (Fig. 6E). This finding indicates that adding additional torque to try to force an over-peptide transition yields ever greater resistance from torsional constraints. In contrast, no such association between torque and unfavored φ/ψ torsions was noted for the under-peptide simulations. While fewer unfavored torsions were observed for the over-peptide simulations at low torques (spring constants of 100–200 kJ/mol/nm^2^), this is explained by the simulation’s resistance to adopting unfavored torsions in the absence of large forces. As torque increases to overcome this resistance, even more torsional strain is encountered relative to the under-peptide simulations, and the peptide still does not flip.

### The limbo dance flipping mechanism is sterically hindered in the WT peptide due to the motions in the WT peptide/HLA-A3 complex

We used the same SMD procedure that illuminated the flip in the neoantigen to examine rotation of the WT peptide, beginning with the structure of the TCR-free peptide/HLA-A3 complex and applying force to rotate the pTrp6 side chain into the position seen when bound to TCR4. Using spring constants as high as 1600 kJ/mol/nm^2^, the side chain would not adopt the TCR-bound state in an over-peptide rotation, again due to unfavored φ/ψ torsions (Fig. 6D, **WT over**). We also could not force an under-peptide transition, again with spring constants as high as 1600 kJ/mol/nm^2^. Investigating why, we found that the higher mobility of the pHis2 side chain allowed it to move into the path of the rotation, sterically blocking the transition due to the formation of contacts and even van der Waals overlap between atoms of the pTrp6 and pHis2 side chains (Fig. 6F, **left**). This was not seen with the neoantigen, as the less mobile pLeu2 remained distant from the pTrp6 side chain (Fig. 6F, **right**). These results are consistent with the unbiased traditional MD simulations, which for the WT peptide showed contacts and the potential for steric interference as pTrp6 entered the base of the groove, but much fewer contacts and potential for interference with the neoantigen (Figs. 5F,G).

### Experimental confirmation that the identity of the position 2 anchor alters the motions of pTrp6 in the HLA-A3 binding groove

**Extended Data Figure 6.**
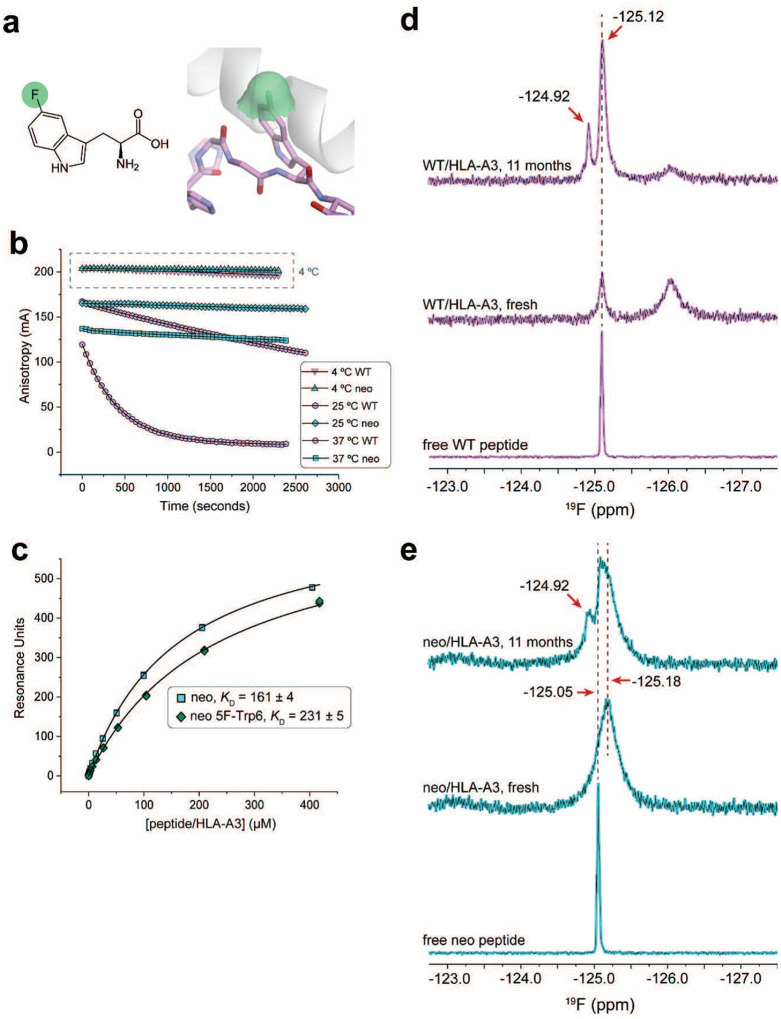
Fluorine modification of pTrp6 in the neoantigen and WT peptide/HLA-A3 complexes and the impact of sample degradation over time. **A)** 5-fluoro-tryptophan and its incorporation in the peptide/HLA-A3 complexes. The fluorine is exposed when modeled in the static structures, as indicated by its solvent accessible surface in the complex with the WT peptide (green surface). **B)** Neoantigen and WT peptide dissociation kinetics from HLA-A3 as a function of temperature, measured by fluorescence anisotropy. While the more rapid dissociation of the WT peptide is evident at higher temperatures, at 4 °C the dissociation kinetics are extensively slowed (37 °C data from ref. (15). **C)** The fluorine modified neoantigen is compatible with TCR binding, as indicated by the binding of TCR3 to the 5F-Trp modified neoantigen/HLA-A3 complex. **D)** The −125.12 ppm resonance in the WT peptide/HLA-A3 complex is from sample degradation, as indicated by comparison of a fresh sample with a sample stored at 4 °C for approximately 11 months, in which the sharp −125.12 ppm resonance is substantially increased and the other major resonances diminished. The −125.12 ppm resonance matches the position of the ^19^F in the free WT peptide yet has a broader linewidth, indicating residual interaction with the protein. In addition, another resonance at −124.92 ppm has emerged, reflecting further degradation of the complex. **E)** The new resonances emerging in an aged sample of the neoantigen/HLA-A3 complex. After 11 months, the peak at −125.18 has developed a shoulder at the position of the free peptide (−125.05 ppm), and a peak has emerged at approximately −124.92 pm. This indicates a degradation pathway similar to that of the WT complex, although degradation is less pronounced, consistent with the greater stability of the neoantigen/HLA-A3 complex.

The molecular dynamics simulations indicate that our inability to detect TCR recognition of the WT peptide/HLA-A3 complex emerges from how the position 2 amino acid influences the motions of the peptide in the HLA-A3 binding groove, with the WT sample possessing greater dynamic behavior but a hindered ability to transition underneath the peptide backbone to the TCR-bound, flipped state. To confirm that the identity of the position 2 amino acid indeed alters peptide motions, we used ^19^F nuclear magnetic resonance (NMR) spectroscopy, replacing the pTrp6 in the neoantigen and WT peptide with 5-fluoro-tryptophan (5F-Trp), which in the static crystallographic structures of the neoantigen and WT peptide/HLA-A3 complexes would leave the fluorine atom solvent exposed (Extended Data Fig. 6A). The fluorine chemical shift is highly sensitive to local molecular environment due to the paramagnetic shielding caused by the lone-pair electrons of the fluorine atom (45–47). Although structural interpretations of fluorine spectra emerging from the complex local environments in proteins is notoriously difficult, comparative analyses can yield insight into conformational and dynamic differences (48, 49).

Anticipating lengthy NMR data collection times, we first verified the stability of the peptide/HLA-A3 complexes. We previously used variants of the *PIK3CA* neoantigen and WT peptides fluorescently labeled at position 5 to monitor peptide dissociation rates via fluorescence anisotropy (15). We repeated those experiments here but expanded the temperature range to include 25 °C and 4 °C in addition to our previously reported 37 °C data. Although the WT complex was markedly less stable at 37 °C and 25 °C, stability was enhanced at 4 °C, with only a small amount of dissociation observed over an extended period (Extended Data Fig. 6B). This result gave us confidence to proceed with NMR experiments, but still cautioned us about the potential for sample degradation as described below. To verify that the presence of the fluorine atom does not fundamentally alter the behavior of the system, we investigated TCR binding to the 5F-Trp substituted neoantigen/HLA-A3 complex. Using SPR, we measured a *K*_D_ for TCR3 that was very close to that measured with the unlabeled neoantigen complex, indicating that fluorine on pTrp6 does not alter the ability of the peptide to flip or significantly destabilize the TCR-bound state (Extended Data Fig. 6C).

**Figure 7.**
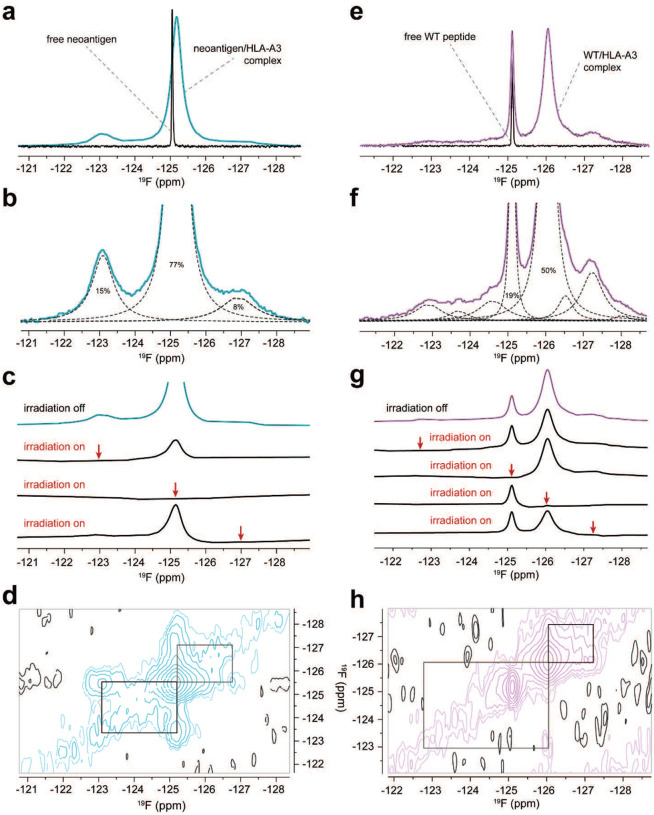
Experimental confirmation of differential peptide dynamics in the HLA-A3 binding groove through ^19^F NMR. **A)** One-dimensional ^19^F NMR spectra of the free neoantigen and the neoantigen/HLA-A3 complex. Whereas the spectrum of the free peptide shows a single sharp peak, the spectrum of the complex shows the presence of multiple states, with broad linewidths as expected for the 45 kD complex. Note that the resonance frequency of the free peptide and the major form of the neoantigen/HLA-A3 complex do not coincide. **B)** Zoomed in view of the neoantigen/HLA-A3 complex, with relative peak areas as determined by line shape fitting (dashed lines). **C)** Conformational Exchange Saturation Transfer (CEST) experiments demonstrating that the multiple peaks in the neoantigen/HLA-A3 complex result from the ^19^F spin experiencing a slow dynamic exchange between at least three different conformations. All four traces were acquired with the same receiver gain, and positions of selective irradiation are shown by red arrows. Irradiation of each of the major resonances significantly reduced the intensity of the two other peaks indicating that they arise from the same ^19^F spin dynamically switching between distinct environments. **D)** Two-dimensional Exchange Spectroscopy (EXSY) experiments independently confirm all three peaks of the neoantigen complex are in conformational exchange. Black rectangles indicate positions of cross-peaks expected if the diagonal peaks represented alternative environments of ^19^F in dynamic equilibrium in a slow exchange regime. The mixing time of the experiment was 50 ms. **E)** As is in panel A but for the free WT peptide and the WT peptide/HLA-A3 complex. Note the coinciding but different widths of the resonance of the free peptide and a major peak for the peptide/HLA-A3 complex, as well as the more complex pattern of additional peaks for the WT complex compared to the neoantigen complex shown in panel A. **F)** As in panel B but zoomed in for the WT peptide/HLA-A3 complex. Line shape fitting reveals at least eight peaks, compared to the three with the neoantigen complex in panel B. Excluding the sharper peak at −125.12 ppm, the linewidths of these peaks are similar those of the neoantigen complex and consistent with a large 45 kD complex. **G)** As in panel C but for the WT peptide/HLA-A3 complex. Selective irradiation at each resonance reduced the intensity of the others except for the resonance at −125.12 ppm. Likewise, selective irradiation at −125.12 ppm saturated this resonance but did not alter the intensity of the other peaks. The multiple broad peaks thus represent states in slow conformational exchange on the NMR time scale with exception of the −125.12 ppm resonance, which corresponds to a distinct, non-exchanging but still protein-bound population of the peptide. **H)** As in panel D, but for the WT peptide/HLA-A3 complex, showing cross-peaks for major resonances except for that at −125.12 ppm. The lower signal-to-noise ratio in the WT peptide/HLA-A3 sample lead to poorer detection of some cross-peaks, yet still confirms conformational exchange between at least three conformational states as well as the non-exchanging character of the −125.12 ppm resonance.

We examined one-dimensional ^19^F spectra of freshly purified samples of HLA-A3 presenting either the neoantigen or wild-type peptide. All data were collected at 5 °C to promote sample stability. One-dimensional ^19^F NMR spectra of the free peptides under the same conditions indicated the chemical shifts and linewidths of the fully hydrated fluorine in the free peptides, which were −125.05 ppm and −125.12 ppm for the neoantigen and WT peptide, respectively (Figs. 7A,E). The spectrum for the HLA-A3 complex with the neoantigen featured an upshifted major peak at −125.18 ppm (Fig. 7A). The fluorine signals from the complex were significantly broader than those of the free peptide, indicating that the ^19^F atom experienced the slow rotational diffusion expected for the 45 kDa peptide/HLA-A3 complex. Closer examination of the spectrum revealed two additional minor peaks at −123.09 ppm and −126.90 ppm with the total signal areas split among the three peaks as 15%, 77%, and 8% (Fig. 7B). We interpreted the major peak at −125.18 ppm as originating from a fluorine accessible to solvent yet tightly associated with the protein. The two minor peaks at −123.09 ppm and −126.91 ppm were also broadened, indicating two additional states with distinct environments for the fluorine atom.

To establish that the three peaks in the neoantigen complex reflected interconverting conformations of the pTrp6 side chain, we performed chemical exchange saturation transfer (CEST) experiments. These experiments confirmed that all three states in the neoantigen/HLA-A3 complex were in slow conformational exchange, as selective pre-saturation of any of these three peaks significantly reduced the intensity of the remaining two signals (Fig. 7C). Two-dimensional exchange spectroscopy (EXSY) experiments independently confirmed that all three peaks in the neoantigen complex originated from the same molecule transitioning between three conformational states, as indicated by cross-peaks connecting the resonance frequencies of the exchanging conformations (Fig. 7D).

By contrast, the spectrum for the WT peptide/HLA-A3 complex was more complex than that with the neoantigen. There were two major peaks, the sharper of which at −125.12 ppm aligned with the position of free peptide and represented about 19% of the total signal (Fig. 7E). A second broader peak was observed upfield at −126.04 ppm and corresponded to roughly 50% of the remaining population. Line shape fitting revealed that the spectrum for the WT peptide/HLA-A3 complex contained several additional peaks from −122.90 ppm to −128.03 ppm (Fig. 7F). The upshifted major peak is consistent with reduced exposure of the ^19^F nucleus to the base binding groove (50), as suggested by our MD simulations, while the greater number of peaks indicated a larger conformational ensemble than seen with the neoantigen. CEST experiments showed that all the peaks were in exchange except for the peak at −125.12 ppm, as selective pre-saturation influenced all but the −125.12 ppm signal, and irradiation at −125.12 ppm did not influence the others (Fig. 7G). This result was confirmed by two-dimensional EXSY, which revealed a lack of detectable cross-peaks with the −125.12 ppm signal (Fig. 7H). As it was sharper and not in exchange, we interpret the peak at −125.12 ppm as resulting from sample degradation, owing to the lower stability of the WT peptide/HLA-A3 complex. We confirmed this by re-collecting data on the same sample after approximately 11 months of storage at 4 °C. The spectrum of this aged complex showed a significant increase in the resonance at −125.12 ppm and a corresponding decrease in the others, along with the emergence of a new resonance at −124.92 ppm (Extended Data Fig. 6D). The fact that the major degradation peak at −125.12 ppm overlapped with but was broader than the signal of free peptide indicates that in the degraded state, the fluorine atom experiences a local environment similar to that in the free peptide, yet the peptide is tumbling slowly and likely still associated with HLA-A3 heavy chain.

The spectrum of a similarly aged neoantigen/HLA-A3 complex was less distorted but did show the emergence of a peak at −124.92 ppm and a shoulder at −125.05 ppm, indicating a very similar but less populated degradation pathway, as would be expected given the higher stability of the neoantigen/HLA-A3 complex (Extended Data Fig. 6E). Thus, its lower stability notwithstanding, the WT peptide/HLA-A3 complex shows more dynamic complexity than that of the neoantigen, confirming that the identity of the position 2 amino acid influences structural and dynamic properties in the center of the peptide.

## Discussion

A defining feature of TCRs is their inherent cross-reactivity, a biological necessity that emerges from a relatively constrained T cell repertoire relative to the large universe of potential epitopes, the structural features of both the TCR and its peptide/MHC ligand, as well as the weak-to-moderate binding affinities that characterize TCRs post-thymic selection. Long held estimates place the average number of peptide/MHC ligands that are compatible with a given TCR as high as a million (1, 2). Remarkably though, TCRs can also show very high specificity, detecting subtle changes in the composition of peptides. While this can often be explained using well known structure/activity relationships (for example, changes to peptides that alter hotspots in the interface or significantly change peptide conformation) (3), TCRs often show surprising and unpredictable sensitivity that cannot be easily rationalized, even when high resolution structural information is available. This sensitivity complicates numerous efforts in immunology that involve TCR specificity, ranging from the selection and engineering of vaccine candidates to predicting viral epitopes and understanding mechanisms of escape mutations.

Here we studied the capacity for TCRs to distinguish between optimal and suboptimal primary anchors in a public neoantigen resulting from a recurrent hotspot mutation in the driver oncogene *PIK3CA*. TCR sensitivity to anchor modification, separate from the impacts on peptide binding to MHC, is well recognized (5, 8–13). In prior work, we suggested that differences in peptide anchor residues could modulate the motional properties of the peptide and MHC binding groove, impacting TCR recognition via dynamic allostery, in which protein motions propagating from changes at one site influence the behavior at another site (19, 20). Although difficult to demonstrate as it often manifests without obvious changes in static structures, dynamic allostery has emerged as a fundamental component of protein behavior, influencing molecular recognition and signaling throughout biology (51). While the sensitivity of the motions of peptide/MHC complexes to peptide modifications is well established (10, 52–55), clear demonstration of peptide/MHC dynamic allostery modulating TCR specificity has been lacking.

Our results show unambiguously that dynamic allostery controls the specificity of TCRs to a mutation-induced anchor modification in the *PIK3CA* neoantigen presented by HLA-A3. The mechanism relies on motions intrinsic to the peptide/HLA-A3 complexes, with rapid movements of the histidine at position 2 of the WT peptide hindering a large and slower conformational flip in the tryptophan at position 6 that is required for TCR binding. The hindrance from the WT peptide evokes a dynamic gate, and only emerges because the path the tryptophan 6 must follow as it transitions across the HLA-A3 peptide binding groove takes it underneath the peptide backbone, where high frequency motions initiated by the suboptimal position 2 anchor interfere. The path the tryptophan takes is in turn facilitated by the presence of a large volume of empty space underneath the peptide backbone, which the neoantigen but not WT peptide is able to sample.

One unresolved mechanistic question relates to why the tryptophan in the *PIK3CA* neoantigen must flip across HLA-A3 upon TCR binding, as space is seemingly available in the base of the HLA-A3 binding groove. One clue is found in the conformations sampled: while the tryptophan mostly samples space in the base of the groove, it has a propensity to reach up and out, where it would be incompatible with the structure of an incoming TCR. As the TCR closes in, attracted by complementary features distributed across the surface of the peptide/HLA-A3 complex (56–58), available space above the peptide groove is reduced. We hypothesize that this reduction, possibly along with expulsion of bulk solvent, destabilizes the conformation of the tryptophan alongside the α1 helix, and it thus takes advantage of the space under the peptide backbone and rotates underneath and around to the α2 helix side of the groove, where it can form new stabilizing interactions that help offset the energetic cost of the flip. A partial flip, with the tryptophan remaining in the base of the groove, is seemingly unable to be stabilized. Related to this, a hypothetical over-peptide flip, beyond being incompatible with peptide torsions as we show, would be incompatible with the initial and transitory TCR-peptide/HLA-A3 contacts formed during the TCR binding process, preventing the receptor from binding at all.

The mechanism of distinction between the *PIK3CA* neoantigen and its WT counterpart helps explain the neoantigen’s strong immunogenicity *in vivo*, at least with the T cells studied (15). The inability of the WT peptide to adopt a conformation compatible with TCR binding renders the neoantigen and WT peptide antigenically distinct, ensuring central and peripheral tolerance mechanisms that operate on the WT peptide are inoperable on the neoantigen (59). With increasing efforts to identify immunogenic cancer neoantigens, understanding how neoantigens overcome tolerance is growing in importance. This is particularly relevant given recent findings that neoantigens that bind MHC proteins with extremely weak affinities can still be potent drivers of tumor immunity (60–62). Although there are multiple mechanisms described by which neoantigens overcome self tolerance (22), it may be notable that this is not the only case where conformational changes and inherent peptide dynamics contribute to discrimination between a neoantigen and its WT counterpart (25, 63).

While the mechanistic details uncovered here are specific to TCR discrimination between the *PIK3CA* neoantigen and its WT counterpart, multiple aspects of our findings are applicable to TCR specificity in general. As noted above, TCR sensitivity to subtle peptide modifications in the absence of structural changes is well documented, occurring in viral, self, and tumor antigens (5, 9–13). The varied motions of peptides in MHC binding grooves have been shown by multiple experimental techniques (64, 65), as have the ability of different peptides to alter the dynamics of MHC proteins (10, 52–55). As we quantify here, conformational changes in peptides upon TCR binding are commonly seen, as are open volumes within the peptide binding groove. Although they have not been studied in as much detail, other examples where structurally silent TCR specificity has been observed have indicated differential peptide/MHC dynamics (5). We thus suggest that dynamic allostery is common in TCR recognition of peptide/MHC, emerging from the architecture of peptide/MHC complexes (particularly for class I proteins) and the dynamic nature of proteins in general. We suggest that this process, which notably occurs within the ligand of the TCR, is a key component to specificity in T cell recognition, reflecting the cooperative evolution of a receptor-ligand sensing system that must simultaneously possess high cross-reactivity and high specificity. While accounting for dynamic allostery in predicting T cell specificity is likely to be challenging, the growing appreciation of how protein motions impact molecular recognition across biology, combined with ever-more rapid development of structural modeling and prediction tools, is catalyzing the development of predictive approaches that are likely translatable to recognition in cellular immunity (66).

An aspect that remains unclear is the effect to which evolution has tuned the capacity for dynamic allostery in peptide/MHC complexes. Although the many thousands of class I MHC variants are structurally homologous, the roles of the underlying MHC polymorphisms that are distributed throughout the peptide binding groove have not all been clarified (67). While their impact on peptide selection and other features such as protein stability are well appreciated (68), in other cases they have also been shown to directly or indirectly impact TCR recognition, not always in structurally visible ways (69–75). As has been shown by single mutation studies (55), it is thus possible that evolution has selected for polymorphisms that tune MHC energy landscapes, allowing for differential dynamic responses to peptides and further enhancing the diversity and complexity of antigen presentation and recognition in cellular immunity.

## Methods

### Recombinant protein preparation

The *PIK3CA* neoantigen, wild-type peptide, Bta, and 5F-Trp variants were purchased from GenScript at >80% purity and dissolved in DMSO prior to refolding. Proteins, including peptide/MHC complexes, TCRs, and the s3–4 scTv were purified from bacterially expressed inclusion bodies from as previously described (15). Briefly, the extracellular domain of the MHC heavy chain, β_2_-microglobulin, TCR α and β chains, and the s3–4 scTv were overexpressed in *E coli* and the resulting inclusion bodies solubilized in 8 M urea and 6 M guanidine-HCl. Denatured proteins were refolded in either peptide/MHC refolding buffer (400 mM L-arginine, 100 mM Tris-HCl, 2 mM NaEDTA, 6.3 mM cysteamine, 3.7 mM cystamine and 0.2 mM PMSF, pH 8.3) or TCR refolding buffer (50 mM Tris-HCl, 2.5 M urea, 2 mM Na_2_EDTA, 6.5 mM cysteamine, 3.7 mM cystamine and 0.2 mM PMSF, pH 8.15) at 4 °C and incubated overnight. The refolding buffer was then dialyzed against ultrapure H_2_O followed by 10 mM Tris-HCl (pH 8.3) at room temperature (for peptide/MHC) or 4 °C (for TCR and scTv) for 48 hours. The refolded proteins were subsequently purified by anion exchange followed by size exclusion chromatography. Protein concentrations were determined by UV absorbance using sequence-determined extinction coefficients.

### X-ray crystallography

The Bta-substituted neoantigen/HLA-A3 complex was solubilized in 10 mM HEPES, 20 mM NaCl, pH 7.4 prior to crystallization. Crystals were obtained by hanging drop vapor diffusion at 4 °C from 15% w/v polyethylene glycol 3350 and 150 mM CsCl. Crystals were cryoprotected with 15–25% glycerol prior to flash-freezing in liquid nitrogen. X-ray diffraction data were collected at the 24-ID-C beamline of the Advanced Photon Source at Argonne National Laboratory. Diffraction data were processed through HKL2000 (76) and initially phased by molecular replacement using Phaser in Phenix (77). The search model was PDB 7L1C with the peptide removed. The peptide was then manually rebuilt in Coot after the model was obtained from Phenix AutoBuild. The model was further refined automatically in Phenix and manually in Coot (78). The composite/iterative build OMIT map was calculated with simulated annealing using CNS as implemented in Discovery Studio 2023. Structures were visualized and analyzed in PyMOL and Discovery Studio.

### Structural analyses and comparisons

Solvent accessible surfaces and surface areas were calculated in either Discovery Studio or VMD (79) with a 1.4 Å radius probe. Continuum electrostatic calculations were performed with H++ using default options (80). The *pK*_a_ of HLA-A3-bound pHis2 was computed using the crystallographic peptide/HLA-A3 structure (PDB 7L1B); the *pK*_a_ of the free peptide was computed using conformation of the peptide from PDB 7L1B with the atoms of the heavy chain and β_2_-microglobulin removed. Electrostatic destabilization was calculated from *pK*_a_ values using ΔΔG°=−2.303*RT*(*pK*_a,bound_ −*pK*_a, free_), where *R* = 1.987 cal/K/mol and *T* = 298.15 K. Cavities in peptide/MHC complexes were quantified with Caver Analyst 2.0 with default options except pockets were excluded, recording the summed volumes of the largest contiguous pockets between the peptide and the HLA-A binding groove (81). Complexes with cleaved, covalently bonded, or non-peptidic ligands were excluded. MoloVol 1.1 was used for visualization of the cavity in Extended Data Fig. 4E (82). TCR-free/TCR-bound RMSD calculations for nonameric peptides presented by class I MHC proteins were calculated via UCSF Chimera (83), identifying all pairs of identical nonamers in the PDB as of March 24, 2024. For instances in which peptides were represented by multiple, replicate structures, we only included the comparison that yielded that yielded the largest Cα or all atom RMSD. In cases where there was a numerical mismatch of atoms between pairs of peptides due to unresolved atoms, atoms were stripped from the fully resolved peptide to match the peptide with missing atoms. As structures exist for some peptides in complex with multiple TCRs, the analysis yielded 67 comparisons for 39 nonameric peptides. For both RMSD and cavity calculations, when multiple molecules were present in crystallographic asymmetric units, only the first molecules were used.

### Binding measurements

Affinities and binding kinetics were measured via surface plasmon resonance using a Biacore T200 instrument. Proteins were buffer exchanged into HBS-EP buffer (10 mM HEPES, 150 mM NaCl, 3 mM EDTA, 0.005% surfactant P20, pH 7.4) prior to experiments. The s3–4 scTv was employed as a positive control and the irrelevant Tax_11–19_/HLA-A*02:01 or gp100/HLA-A*02:01 complexes as a negative control. For affinity measurements, TCRs and s3–4 were immobilized on a CM5 Series S sensor chip (Cytiva) to 400–6000 RU via amine coupling and peptide/MHC complexes were injected at a flow rate of 5 μL/min. Experiments were performed at either 4 °C or 25 °C as indicated with a blank activated and deactivated flow cell as reference. Binding affinities were determined by fitting the curve of the reference-subtracted steady-state responses against the injected protein concentrations to a 1:1 binding model in OriginPro 2024. For measurements of dissociation rates, experiments were performed at 4 °C. TCRs or the s3–4 scTV were immobilized to approximately 300 RU on a CM5 Series S sensor chip with peptide/MHC complexes injected at a flow rate of 100 μL/min. The dissociation rate (*k*_off_) of a TCR-pMHC binding was determined by fitting the dissociation phase of the sensorgram to a single exponential decay function in OriginPro 2024. The association rate (*k*_on_) was calculated from the ratio of measured *k*_off_ and the separately measured *K*_D_ at 4 °C using *k*_on_=*k*_off_/*K*_D_.

### T cell functional analysis

The peptide specificity of TCR-transduced T cells was assessed by intracellular cytokine staining (ICS) using the BD Cytofix/CytoPerm Plus Kit, following manufacturer’s instructions. Cos-7 cells were electroporated with 100 μg/mL of HLA-A*03:01 mRNA and plated into 96-well round-bottom plates overnight. Cells were pulsed with titrating amounts of indicated peptides for 30 minutes at 37 °C. Cells were washed with 1x PBS to remove any unbound peptide. TCR-expressing T cells were co-cultured at an E:T ratio of 1:1 for 6 hours in the presence of anti-CD107A- BV650 (Clone H4A3, BioLegend) and Golgi block. Cells were washed in 1× PBS and surface labeled with Live/Dead fixable dye (Invitrogen), anti-CD3-APC-H7 (Clone SK7, Invitrogen), anti-CD8-efluor450 (Clone SK1, Invitrogen) and anti-mouse TCR-PerCpCy5.5 (Clone H57–597, Invitrogen) for 30 minutes at 4 °C. Cells were washed with 1×PBS and then fixed and permeabilized for 15 minutes at 4 °C. Surface-labeled cells were then washed with 1× perm-wash buffer and labeled with anti-TNFα-PE (Clone Mab11, Invitrogen) for 30 minutes at 4 °C in perm-wash buffer. All antibodies were used at a final concentration of 5 μg/mL. Finally, cells were washed with perm-wash buffer, suspended in 2% FBS in PBS and acquired on an X20 LSR Fortessa flow cytometer with the BD FACSDiva software. Data were analyzed using FlowJo software version 10.6.2.

### Differential scanning fluorimetry

Thermal denaturation of peptide/HLA-A3 complexes was performed using differential scanning fluorimetry using a Prometheus NT.48 (NanoTemper) monitoring intrinsic tryptophan fluorescence as previously described (18). Briefly, 10 μL of neoantigen/HLA-A3 and Bta-substituted neoantigen/HLA-A3 at concentrations of 15 μM in HBS-EP buffer (10 mM HEPES, 150 mM NaCl, 3 mM EDTA, 0.005% surfactant P20, pH 7.4) were loaded into instrument capillaries. The temperature was scanned from 20 °C to 95 °C at a constant rate of 1 °C/min. Fluorescence at emission wavelengths of 330 nm and 350 nm were recorded and the first derivative of ratio of the fluorescence intensities were plotted vs. the temperature to generate the melting curve and the data fit to bi-Gaussian functions using OriginPro 2024.

### Traditional molecular dynamics simulations

**Extended Data Figure 7.**
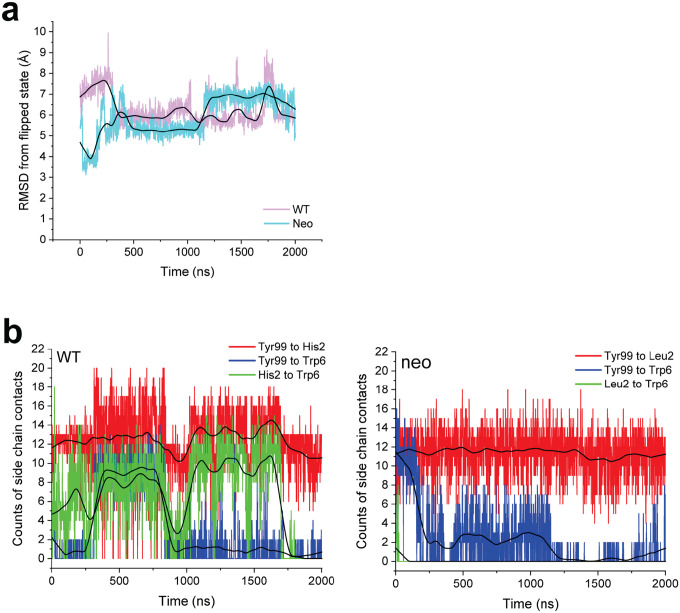
Unsmoothed RMSD and interaction count data. **A)** Unsmoothed RMSD values for the pTrp6 amino acid relative to its conformation in the ternary complex with TCR4, indicating that side chain does not flip in either peptide/HLA-A3 simulation. Solid black lines show the LOWESS smoothed data shown in Fig. 4B. **B)** Unsmoothed counts of side chain/side chain contacts for pHis2/pLeu2, pTrp6, and Tyr99 of HLA-A3 as a function of time during the two simulations. More contacts are made in the WT simulation, particularly for position 2-pTrp6 and Tyr99-pTrp6. Solid black lines show the LOWESS smoothed data shown in Extended Data Fig. 3C.

Fully atomistic, unrestrained molecular dynamics simulations (referred to as traditional MD) were performed on GPU hardware with Amber18 using the ff14SB force field and an SPC/E water model as previously described (84). Structures of the *PIK3CA* WT peptide/HLA-A3 complex (PDB 7L1B), *PIK3CA* neoantigen/HLA-A3 complex (PDB 7L1C), and TCR4-bound neoantigen/HLA-A3 complex (PDB 7L1D) with the TCR removed were utilized as the starting coordinates for each simulation. Peptides were modeled with charged terminal residues. Sodium ions were added as the counterions to neutralize the system. After initial energy minimization, systems were heated to 300 K with Langevin dynamics. Solute restraints were gradually relaxed under constant pressure from 25 to 0 kcal/mol/Å. 50 ps of NVT simulation was then performed, followed by production simulations. Production trajectories were calculated under constant volume with a 2 fs time step for 2 μs. Analyses of trajectories were carried out with CPPTRAJ (85). Mass weighted amino acid RMS fluctuations were calculated via the CPPTRAJ ‘atomicfluct’ command. 1D RMSD values were calculated via the CPPTRAJ ‘rms’ command after superimposition the Cα atoms of the HLA-A3 binding groove (residues 1–180). D-score values were calculated as previously described (28, 29) using the average ψ/φ angles of peptide residues obtained via the CPPTRAJ ‘dihedral’ command. Grid space occupancies of peptide residues were calculated via combinatorial usage of the CPPTRAJ ‘bounds’ and ‘grid’ command using a grid spacing of 0.1 Å. Occupied grid space was visualized through the volume viewer in USCF Chimera. The grid space volume was first smoothed via a Gaussian filter and contoured to encompass grid space occupied for at least 10% of simulation time. Side chain torsion angles and hydrogen bonds were calculated via the Chimera ‘dihedral’ and ‘hbond’ commands, respectively. Conformational clustering of peptide residues was carried out in MATLAB R2022a and was performed on 2D-RMSD matrices calculated via the CCPTRAJ ‘rms2d’ command from Cα superimpositions of the HLA-A3 binding grooves (residues 1–180). The matrices were calculated in two different formats depending on the residue being investigated. For the position two anchor residue, because the side chain differs between the two peptides, independent matrices were generated for the neoantigen and WT simulations, each comprised of the simulation data and the respective initial crystallographic coordinates (PDB IDs 7L1B and 7L1C). For p6Trp, a single matrix was generated which was comprised of the simulation data of both the neoantigen and WT simulations, both sets of TCR-free crystallographic coordinates (PDB IDs 7L1B and 7L1C), and the crystallographic coordinates of the ternary complex with TCR4 (PDB ID 7L1D). The optimum number of clusters for each system of interest was calculated with the MATLAB ‘evalclusters’ command utilizing agglomerative clustering and the Calinski-Harabasz Index (86). Dendrograms of the clustered 2D-RMSD data were generated via the MATLAB ‘clustergram’ command. Average structures of each cluster were generated via the CCPTRAJ ‘average’ command. Clusters were visualized by averaging the coordinates in the simulation frames representing each cluster. RMSD data in Fig. 4B and side chain contact counts in Extended Data Fig. 3C were smoothed using locally weighted scatterplot smoothing (LOWESS) as implemented in OriginPro 2024 using default options (unsmoothed data in Extended Data Fig. 7). Interatomic contacts were defined as interatomic distances < 4 Å; hydrogen bonds were determined with a donor-acceptor distance cutoff of 3.5 Å.

### Weighted ensemble molecular dynamics simulations

Weighted ensemble molecular dynamics simulations of the neoantigen/HLA-A3 complex were performed via the Weighted Ensemble Simulation Toolkit with Parallelization and Analysis (WESTPA) package (41, 42). For forward simulations, starting coordinates were the neoantigen/HLA-A3 complex (PDB 7L1C) and the target was the position of pTrp6 in the TCR4-bound A3 ternary complex (PDB 7L1D). This was reversed for the reverse simulation, with the coordinates of the TCR removed for the starting coordinates. The simulations were initiated from statistically independent configurations separated by 200 ns from traditional MD production simulations initiated and performed as described above. Full-atom RMSDs between pTrp6 in the neoantigen in the simulation and its position in the corresponding target coordinates were utilized as progress coordinates. The starting value of the progress coordinate was 6.39 Å for the forward simulation and 6.51 Å for the reverse simulation. Progress coordinates in both cases were divided into 72 bins, ranging from 0.0 Å to infinity with steps designed to minimize bias in the transition directions recorded by WESTPA. Bin values were 0.0–2.0 Å (7 bins with an even step of 0.3 Å), 2.0–6.0 Å (40 bins with an even step of 0.1 Å), 6.0–11.0 (24 bins with an even step of 0.2 Å) and ≥ 11.0 Å (1 bin). Iteration time was set at 10 ps and the maximum number of trajectories in each bin was 8. All the simulation settings were the same as those for production simulations in traditional MD, except that distance restraints in the form of flat-welled parabolic potentials between opposing residues in the HLA-A3 α1 and α2 helices were applied to ensure that high energy states captured during WESTPA had proper binding groove geometry. Residues and the corresponding lower and upper distance bounds were Gly79-Ile142 (13.0–19.1 Å), Thr80-Ile142 (9.0–17.4 Å), Tyr 59-Arg170 (10.0–18.3 Å), Tyr59-Asn174 (10.0–19.8 Å). Solvent accessible surface area data in Fig. 6C were smoothed using LOWESS as implemented in OriginPro 2024 using default options to generate the indicated curve. A hypothetical over-peptide conformation for computing the surface area in Fig. 6C was generated by manually adjusting the χ1 torsion of pTrp6 in PDB 71LC to 27°, resulting in the side chain pointing directly out of the groove. For generating the Supplemental Movie, the Cα atoms of the HLA-A3 peptide binding groove (residues 1–180) for all 5410 frames of a successful transition were aligned using CCPTRAJ, and the movie generated in Chimera with a step size of 20 frames.

### Steered molecular dynamics simulations

Steered molecular dynamics was performed using enforced rotation (44). Trajectories were generated using GROMACS 2022 with the CHARMM36-jul2021 force field (87, 88). Coordinates of the peptide/HLA-A3 complexes (PDB IDs 7L1B and 7L1C) were solvated with TIP3P water in a dodecahedral unit cell. All systems were charge neutralized by the addition of Na^+^ and Cl^−^ ions to a final concentration of 0.15 M and energy minimized via steepest descent. Short 500 ps of NVT ensemble dynamics were carried out in the presence of heavy atom restraints to stabilize the temperature to 298.15 K with the v-rescale thermostat. Systems were subsequently equilibrated by 1 ns of NPT ensemble dynamics via c-rescale pressure coupling at 1 bar. Restraints were removed and production simulations initiated under the NPT ensemble utilizing Nose-Hoover and c-rescale temperature and pressure coupling, respectively. Rotation groups were defined as all atoms of the pTrp6 amino acid and rotation vectors selected along the peptide backbone. Enforced rotation was carried out in two directions (under and over) for a set of force constants using the flex2-t potential and rotation rate of 0.10°/ps for 500 ps. Force constants used were 100, 200, 400, 800, and 1600 kJ/mol/nm^2^. For the neoantigen rotated under, the subsequent unbiased simulation adhered to the same simulation conditions except without enforced rotation parameters. RMSD measurements of rotated pTrp6 conformations relative to the TCR4-bound conformation (from PDB 7L1D) were obtained from VMD following a superposition of the Cα atoms of the residues 1–180 of the HLA-A3 peptide binding grooves and selection of all pTrp6 atoms. The van der Waals (vdW) overlap between pTrp6 and p2His/p2Leu were computed by PyMOL’s ‘overlap’ command, with overlap for a pair of atoms determined by the difference between the atomic distance between them and the sum of their respective vdW radii. Peptide φ/ψ torsions from enforced rotation trajectories were measured using the MDAnalysis Python library (89). Allowances for side chain torsion angles were determined by first collecting the φ/ψ values for peptide residues and plotting the associated Ramachandran profiles. Frames containing instances outside of allowable regions were counted for peptide amino acids excluding glycines. Allowances were set by the Ramachandran reference data internal to the MDAnalysis library (Rama_ref), which defines allowed and generously allowed regions with boundaries containing 90% and 99% of the reference measurements (90).

### Fluorescence anisotropy

Fluorescence anisotropy measurements for peptide dissociation were performed as previously described (15). Briefly, for both the WT and neoantigen peptides, pGly5 was substituted with a 5-carboxyfluorescein-modified lysine. Experiments were performed on a Beacon 2000 fluorescence polarization instrument at 4 °C and 25 °C by mixing 100 nM fluorescein-labeled peptide/HLA-A3 complexes with an excess of 100 μM unlabeled peptide in 20 mM NaH_2_PO_4_, 75 mM NaCl, pH 7.4. The excitation wavelength was 488 nm and polarization was detected at 535 nm. Changes in anisotropy were recorded as a function of time. Dissociation kinetics of peptides were determined by fitting the anisotropy curve to a single or biphasic dissociation function in OriginPro 2024.

### Nuclear magnetic resonance

^19^F NMR spectra were recorded using Bruker 600 MHz NMR instrument with a QCI cryogenic probe at NMRFAM, University of Wisconsin-Madison. Protein solutions were prepared in 10 mM HEPES, 150 mM NaCl, pH 7.4 with 10% D_2_O at concentrations of 0.5 mM and 0.3 mM for neoantigen/HLA-A3 and WT peptide/HLA-A3 complexes, respectively. To obtain similar sensitivities on the two samples at different concentrations, the total acquisition times of the 1D experiments in Figs. 7A–F in were set to approximately 12 hours and 33 hours, accordingly. The spectra were acquired in 4-hour blocks interleaved with proton 1D recordings to verify sample stability. For the data in Extended Data Figs. 6D,E total acquisition times were 20 minutes for both complexes. Probe temperature was maintained at a calibrated value of 5.2 °C. Once all blocks were recorded, they were checked individually for frequency drift, which was not detected, and combined to obtain combined datasets with improved signal/noise ratio. The reference frequency of the ^19^F channel was set through internal routines of the Topspin acquisition software after the NMR spectrometer was locked on D_2_O. Despite the high concentrations, the large molecular weight of the peptide/HLA-A3 complexes (45 kDa) combined with the fast transverse relaxation of the ^19^F nuclear spin resulted in low detection sensitivity. To improve the signal/noise ratio, we compared recording of ^19^F 1D spectra with and without proton decoupling. Decoupling had a detrimental effect on sensitivity; therefore all spectra were collected undecoupled. We also optimized a recycle delay for the fluorine 1D experiment and determined that 1 s was the optimal time. Therefore, D1 of 0.4 s was used with AQ of 0.59 s. Line shape deconvolution was performed using Mestrelab MNova NMR. Free induction decays were processed with an exponential window function, Fourier- transformed, and baseline corrected prior to fitting with Lorentzian line shapes. CEST experiments were performed by applying 10–40 μW irradiation on a proton channel at a desired ppm position for 0.4 s. The total acquisition time for each CEST dataset was 2 hours. The bandwidth of the saturation pulse was directly measured on a free peptide sample by varying the saturation ppm position around the sharp peptide peak. The direct effect of the pre-saturation pulse was found detectable within +/− 0.5 ppm from the irradiation position. Exchange Spectroscopy (EXSY) experiments were performed with 50 ms mixing time. The neoantigen/HLA-A3 dataset was collected with NS=1024, TD1=32 and SW=10.5 ppm for 10 hours. The WT peptide/HLA-A3 dataset had a weaker signal therefore the SW and TD1 were modified to increase the total number of scans for greater sensitivity: NS was set to 5120 with TD1 = 16 and SW = 6 ppm. Data were collected in four six-hour blocks that were combined for processing. To observe possibly slower exchange dynamics for the non-exchanging sharper resonance in the WT peptide/HLA-A3 sample, we collected an additional EXSY dataset with 200 ms mixing time; no cross peaks were observed confirming an absence of detectable exchange dynamics between the sharp peak and other resonances of WT peptide/HLA-A3 sample.

### Statistics

Quantitative fitting of steady state binding and kinetic dissociation data was performed in OriginPro 2024. Quantitative fitting of NMR lineshapes was performed in Mestrelab MNova NMR. T tests were performed with GraphPad Quickcalcs (https://www.graphpad.com/quickcalcs/ttest1.cfm).

## Extended Data

## Supplementary Material

1
