# Supplementary material for: Dynamic allostery in the peptide/MHC complex enables TCR neoantigen selectivity
Source: Res Sq. 2024 May 29:rs.3.rs-4457195. Preprint. [Version 1] doi: 10.21203/rs.3.rs-4457195/v1 (PMC11160895; doi:10.21203/rs.3.rs-4457195/v1)
Supplement: 1 [file NIHPPrs4457195V1-supplement-1.pdf]

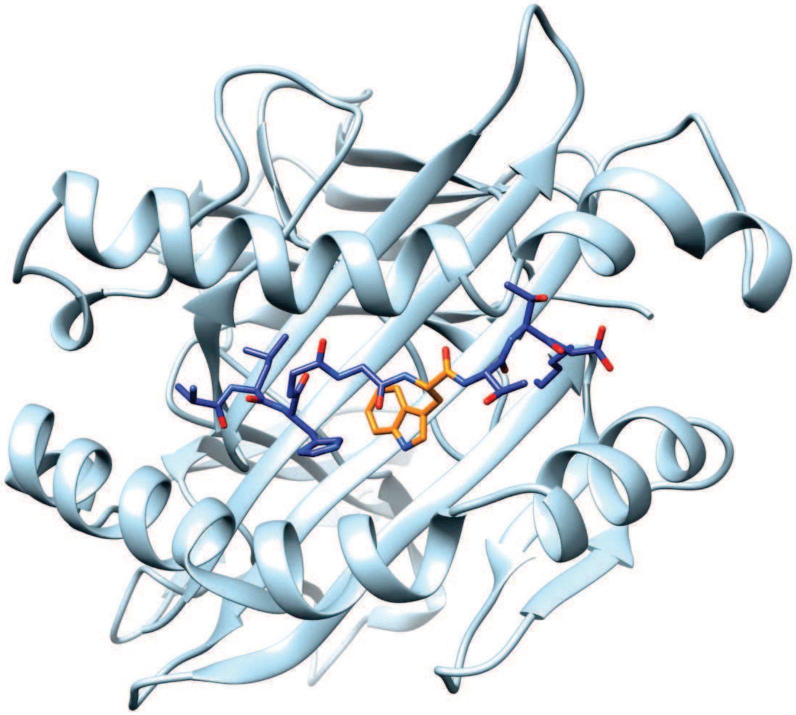

(movie uploaded as a separate file)

Supplemental Movie. Trajectory of an under-peptide neoantigen flip from the reverse WEMD simulations. The trajectory begins with pTrp6 of the neoantigen in the TCR-bound neoantigen (the flipped conformation). It flips via the peptide-limbo mechanism to the TCR-free conformation with a final RMSD to the target state of 0.6 Å. The actual transition is rapid and occurs at approximately 4 seconds, or approximately 2300 frames into the 5410 frame simulation.

Supplemental Table 1. X-ray data collection and refinement statistics for the Bta6-substituted neoantigen/HLA-A3 complex*

	**PIK3CA-Bta6-neo/HLA-A3**
PDB accession code	9ASG
Resolution range (Å)	44.02 - 2.03 (2.10 - 2.03)
Space group	P 6 2 2
Unit cell dimensions (Å)	156.80, 156.80, 85.60
Unit cell angles (°)	90, 90, 120
Total reflections	1,381,818 (105,151)
Unique reflections	40,437 (3563)
Multiplicity	34.2 (26.5)
Completeness	0.972 (0.899)
Mean I/sigma(I)	25.73 (3.72)
Wilson B-factor	24
R-merge	0.202 (1.548)
R-meas	0.205 (1.581)
R-pim	0.036 (0.315)
CC1/2	0.997 (0.778)
CC*	0.999 (0.936)
Reflections used in refinement	39,361 (3563)
Reflections used for R-free	3891 (350)
R-work	0.202 (0.271)
R-free	0.237 (0.302)
CC(work)	0.946 (0.831)
CC(free)	0.936 (0.716)
Number of non-hydrogen atoms	3535
macromolecules	3127
ligands	6
solvent	402
Protein residues	382
RMS(bonds)	0.003
RMS(angles)	0.64
Ramachandran favored (%)	98.66
Ramachandran allowed (%)	1.34
Ramachandran outliers (%)	0
Rotamer outliers (%)	1.82
Clashscore	2.63
Average B-factor ($Å^2$)	39.38
macromolecules	38.95
ligands	36.72
solvent	42.72
Number of TLS groups	4

*Numbers in parentheses are for the highest resolution shell.

Supplemental Table 2. Largest contiguous cavities between peptides and MHC binding grooves in structures of complexes in the HLA-A3 superfamily.

PDB ID	HLA	Peptide length	Volume (Å^3)
2xpg	A3	9	293
3rl1	A3	9	139
3rl2	A3	10	94
6o9b	A3	9	266
6o9c	A3	9	343
7l1b	A3	9	270
71lc (*PIK3CA* neoantigen)	A3	9	401
7mle	A3	9	172
7uc5	A3	9	31
8dvg	A3	10	14
1q94	A11	9	134
1qvo	A11	10	270
1x7q	A11	9	173
2hn7	A11	10	81
4mj5	A11	11	99
4mj6	A11	11	180
5grd	A11	10	80
5gsd	A11	7	267
5wjl	A11	10	53
5wjn	A11	10	342
6joz	A11	9	282
7m8t	A11	9	223
7ow3	A11	10	59
7s8q	A11	10	340
7s8r	A11	9	102
7s8s	A11	10	61
7wkj	A11	9	96
8i5e	A11	9	49
6j1w	A30	9	148
6j1v	A30	9	436
6j29	A30	9	165
6j2a	A30	9	289
4hwz	A68	9	137
4hx1	A68	9	115
4i48	A68	9	151
6ei2	A68	10	70

Average: 178 Å^3
Standard deviation: 113 Å^3
